# Tumour Mutation Burden Does Not Predict Immune Cell Frequency or Risk of Recurrence After Surgery in Hepatocellular Carcinoma

**DOI:** 10.3390/cancers18193079

**Published:** 2026-09-22

**Authors:** Abigail Connor, Leon Chang, Emma J. West, Karen J. Scott, Fay Ismail, Yousef Alghamdi, Alyn Cratchley, Lucy Stead, Darren Newton, Adel Samson

**Affiliations:** 1 Leeds Institute of Medical Research at St. James’s, University of Leeds, Leeds LS9 7TF, UK; a.l.connor@leeds.ac.uk (A.C.); e.j.west@leeds.ac.uk (E.J.W.); k.j.scott@leeds.ac.uk (K.J.S.); f.ismail@leeds.ac.uk (F.I.); umyma@leeds.ac.uk (Y.A.); l.f.stead@leeds.ac.uk (L.S.); d.j.newton@leeds.ac.uk (D.N.); 2 Leeds Institute of Rheumatic and Musculoskeletal Medicine, University of Leeds, Leeds LS7 4SA, UK; l.chang@leeds.ac.uk; 3 Leeds Teaching Hospitals NHS Trust, Leeds LS9 7TF, UK; alyn.cratchley@nhs.net

**Keywords:** hepatocellular carcinoma, tumour immunology, genomic profiling, multifocal cancer, immunotherapy

## Abstract

Introduction/Objectives: Understanding the genetic and immune heterogeneity in hepatocellular carcinoma (HCC) is critical for accurate pathological characterisation and treatment planning, as therapies advance towards personalisation. Tumour mutational burden (TMB) has been proposed as a biomarker of response to immune checkpoint blockade but its relationship with immune infiltration in HCC remains unclear, particularly across different aetiologies and within multifocal disease. Methods: This study characterises both the inter- and intra-patient heterogeneity in HCC, integrating whole-exome sequencing with detailed histopathologic and immune cell profiling, to identify implications for diagnosis and response to treatment. Samples of 54 tumours from 26 patients undergoing surgery at the Leeds Teaching Hospital NHS Trust were examined, including 10 patients with multifocal disease. The cohort includes the major UK aetiological groups. Whole-exome sequencing of tumours and matched background liver identified somatic variants. Histopathological and immunohistochemical evaluations were performed to assess tumour features and characterise the tumour immune microenvironment. Results: Marked inter-individual heterogeneity was observed across aetiological groups with variation in tumour mutation burden, genes containing somatic mutations and immune infiltration. Multifocal tumours frequently demonstrated discordant genetic and immune profiles irrespective of clonal relatedness. NeoTMB was not associated with recurrence following surgery, whereas recurrent cases demonstrated significantly higher intratumoural FoxP3+ T-cell frequencies. Conclusions: This study demonstrates substantial genetic and immune heterogeneity in HCC, including marked discordance between multifocal tumours within the same patient. Tumour mutation burden was not related to immune cell infiltration or risk of recurrence after surgery, whereas elevated intratumoural FoxP3+ regulatory T-cell frequencies were associated with recurrence. These findings suggest that the immunological composition of the tumour microenvironment may be more relevant to clinical outcome than mutational burden alone and highlight the importance of comprehensive tumour profiling to inform personalised therapeutic strategies.

## Simple Summary

Hepatocellular carcinoma is the most common form of primary liver cancer. Tumours can differ considerably between patients and even within the same patient, making it difficult to identify reliable markers that predict tumour behaviour or treatment response. We examined genetic changes, tumour growth and immune cells in liver tumours removed during surgery. In patients with multiple tumours, different lesions frequently had distinct genetic and immune characteristics, including among tumours that shared a common origin. The number of tumour mutations was not associated with immune cell infiltration or recurrence after surgery. In contrast, higher numbers of immunosuppressive FoxP3-positive T cells within tumours were associated with shorter recurrence-free survival, although this finding requires confirmation in larger patient groups. These results suggest that assessing the immune environment of liver tumours may provide information that is not captured by measuring mutation numbers alone. More comprehensive tumour assessment may therefore support the future development of improved biomarkers and personalised treatment strategies.

## 1. Introduction

Hepatocellular carcinoma (HCC) is the third largest cause of cancer-related deaths worldwide, responsible for over 800,000 deaths annually [1] and accounting for approximately 80% of primary liver cancers. Global incidence is projected to increase further, driven by the rising prevalence of underlying risk factors; most commonly by alcohol-related liver disease (ARLD), metabolic dysfunction-associated steatotic liver disease (MASLD) and hepatitis B (HBV)/hepatitis C (HCV) infections [2].

HCC most commonly develops in the context of chronic liver disease or cirrhosis and, consequently, clinical outcome and treatment selection are influenced by both tumour burden and underlying hepatic reserve. Management is stage-dependent and may include surgical resection, liver transplantation or local ablation for early-stage disease, locoregional therapy for intermediate-stage disease and systemic therapy for advanced or unresectable disease [3]. Despite potentially curative treatment, ongoing radiological follow-up is required because of the risk of recurrence. In advanced HCC, immune checkpoint inhibitor-containing combinations have become established first-line treatment options but response rates are limited [4], creating the need for biomarkers that can identify patients most likely to benefit.

HCCs are not defined by a singular genetic event but rather an enrichment of mutations, occurring in specific disease-associated genes. Compared to other tumour types, HCC has an intermediate tumour mutation burden (TMB; [5]), and the literature suggests this is highly variable: [6] reported a range between 0 and 3.5 mutations per megabase. TMB has been linked to immune checkpoint blockade (ICB) response in a meta-analysis of solid cancers, where a 48% reduced risk of disease progression was observed in patients with a ‘high’ TMB, as defined in each study, following ICB treatment [7].

The rationale linking TMB to immunotherapy response is that somatic mutations may generate neoepitopes that drive a T-cell response, leading to anti-tumour immunity [8]. Evidence linking TMB to immune checkpoint inhibition response in HCC is inconsistent. Some transcriptomic analyses have suggested an association between TMB and immune-related gene signatures [9] whilst others report that immune exclusion and immunosuppressive mechanisms may dominate irrespective of mutational burden [10]. Consequently, the extent to which TMB reflects the immune landscape of HCC remains uncertain.

The frequency of tumour-infiltrating lymphocytes is associated with overall survival (OS) in HCC [11], whilst the frequency of the FoxP3+ regulatory subset is instead linked with poorer survival [12]. These observations support the concept that the efficacy of immunotherapy is at least partially dependent on a pre-existing anti-tumour immune response, as seen in non-small-cell lung cancer patients [13].

Whilst several studies have characterised the genetic landscape of HCC according to the underlying aetiological subgroup [14,15], few have compared HCC genetics with the tumour immune microenvironment. Studies looking at individual aetiological groups have observed decreased total T-cell numbers in HCC compared to healthy controls in MASLD-related patients [16]. All aetiologies have been associated with immune cell exhaustion [17] however responses to immunotherapy have been reported to vary between groups, such as being reduced in metabolic dysfunction-related tumours [18]. Characterising the extent of variation in genetic alterations and immune cell frequencies between HCC in this current cohort offers key insights into possible mechanisms of resistance to current immunotherapy treatments, particularly in the more complex setting of multifocal disease. At the time of this study, patients with advanced HCC are most often treated with a combination of atezolizumab and bevacizumab or tremelimumab and durvalumab [19] in the first-line setting, despite only a minority of patients achieving a tumour response, whilst very few have durable responses. Previous studies looking at a multifocal HCC using deep sequencing approaches have identified a high degree of genetic similarity to indicate metastatic disease in cohorts of hepatitis-associated patients [20,21].

It is appreciated that both the genetic heterogeneity and tumour immune microenvironment are relevant for responsiveness to current treatments, yet the literature on the intersection of these is lacking. Previous studies looking at this have focussed on single aetiological groups [21] or have inferred immune cell characteristics from transcriptomic data [20]. The extent to which clonally related tumours diverge with respect to their immune phenotype remains poorly defined at the proteomic and spatial level, yet bears direct relevance for personalised therapies.

In this study, we addressed this gap by performing paired whole-exome sequencing and spatial immunohistochemical profiling across 26 patients, including 10 with multifocal disease, in a UK cohort representing all major aetiological groups.

## 2. Methods

### 2.1. Patient Samples

Archival formalin-fixed paraffin-embedded (FFPE)-preserved tumour and background liver tissues were requested and obtained from the NHS tissue bank via the Department of Cellular Pathology at St James’s University Hospital, subject to the appropriate ethical consent at the point of surgery. Patients undergoing liver transplantation (*n* = 16) or surgical resection (*n* = 10) for HCC between 2013 and 2020 were eligible for inclusion. Patients with mixed hepatocellular-cholangiocarcinoma or fibrolamellar HCC were excluded. Samples were analysed consecutively where tissue and consent were available. The cohort had a median age of 65.5 (range 50–80). The cohort includes ARLD (*n* = 6), MASLD (*n* = 11), HCV (*n* = 2), Haemochromatosis (*n* = 1), and mixed aetiology (*n* = 4). All patients were Barcelona Clinic Liver Cancer stage A at the time of surgery, but some were upstaged to B if more than 3 tumours were identified. No tumours presented with macrovascular invasion. No patient received systemic anti-cancer therapy before surgery. A subset of transplant patients received bridging locoregional therapy (TACE and/or ablation), which is described in Supplementary Table S1, alongside other clinical characteristics.

All research was conducted in accordance with both the Declarations of Helsinki and Istanbul, was approved by the appropriate ethics and institutional review committee. Patients were specifically consented for research use of their explanted liver samples (REC reference 20/NW/0223). Written consent was given in writing by all subjects. Anonymous details can be found in Supplementary Table S1.

### 2.2. DNA Extraction

Sections of FFPE tissue were scraped using a size 22 scalpel blade (Swann-Morton, Sheffield, UK). DNA was extracted using the MagMax FFPE ultra kit (Thermo Fisher, Waltham, MA, USA), including an overnight protease digestion at 55 °C prior to inactivation at 90 °C. Nucleic acids were then quantified using either the Qubit^®^ dsDNA BR Assay Kit or the Qubit^®^ RNA BR Assay Kit on a Qubit^®^ 4 Fluorometer (all Thermo Fisher).

### 2.3. Whole-Exome Sequencing

Genomic DNA was extracted from FFPE liver biopsies and processed using the Exome 2.0 kit (Twist Bioscience, San Francisco, CA, USA). All DNA samples were quantified using the Qubit^®^ 4 Fluorometer before libraries were created and final quality was assessed on the Agilent Tapestation before whole-exome libraries were prepared using the Twist Comprehensive Exome 2.0 kit (Twist Bioscience). Library concentration was assessed using the Qubit^®^ 4 Fluorometer and fragment size distribution was evaluated using an Agilent TapeStation (Santa Clara, CA, USA). Sequencing was performed on the Illumina^®^ NovaSeq™ (San Diego, CA, USA) platform to a mean read depth of 80.9× (range 24.8–164.0×) for tumour samples and 84.0× (range 31.4–159.9×) for matched adjacent normal samples (Supplementary Table S2).

### 2.4. Sequencing Data Processing and Quality Control

Raw paired-end reads were adapter- and quality-trimmed using Cutadapt (Q ≥ 10 at both ends, minimum retained length 30 bp), removing Illumina and poly-G adapter sequences. Read quality was assessed with FastQC both before and after trimming. Sequencing depth and coverage uniformity across the exome target region were evaluated using GATK (v4.3.0.0) DepthOfCoverage against the Twist Comprehensive Exome plus spike-in panel (v2.0.2) target intervals, with per-sample and per-gene summary metrics generated.

### 2.5. Alignment and Pre-Processing

Trimmed reads (from both tumour and matched normal samples) were aligned to the GRCh38 reference genome (UCSC analysisSet build) using BWA-MEM (v0.7.17). Aligned reads were coordinate-sorted and PCR/optical duplicates marked using Picard SortSam and MarkDuplicates (both v3.5.0). Base quality scores were recalibrated using GATK BaseRecalibrator and ApplyBQSR, with known variant sites from dbSNP (build 138) used to model sequencing error covariates, producing analysis-ready BAM files for downstream somatic variant calling.

### 2.6. Somatic Variant Discovery

Somatic single-nucleotide variants and small insertions/deletions were called using GATK Mutect2 (v4.3.0.0) in matched tumour–normal mode, with all tumour replicates for a given patient and their matched normal sample provided jointly, restricted to the Twist Comprehensive Exome target intervals. Mutect2 was run with --genotype-germline-sites true to ensure adequate representation of germline heterozygous positions in the output VCF, a requirement for downstream tumour purity estimation. Calls were informed by a population germline resource (gnomAD allele frequencies) and a panel of normals to suppress recurrent sequencing/mapping artefacts. Cross-sample contamination was estimated for each tumour replicate against its matched normal using GetPileupSummaries and CalculateContamination against a common biallelic SNP panel (ExAC). To account for the characteristic cytosine deamination artefacts of FFPE tissue, read orientation bias was modelled from F1R2 read counts using LearnReadOrientationModel. Final somatic calls were filtered using FilterMutectCalls, incorporating the contamination estimates and orientation-bias artefact priors to exclude FFPE-associated false positives.

### 2.7. Variant Annotation and Filtering

Filtered somatic variants were functionally annotated using the Ensembl Variant Effect Predictor (VEP, GRCh38 cache, v113), including consequence prediction, SIFT/PolyPhen scores, gnomAD population allele frequencies, and CADD deleteriousness scores. Annotated variants were further processed using VASE (Variant Annotation and Segregation Explorer), applying a minimum depth filter (DP ≥ 50), PASS-only filtering, and dbSNP/ClinVar cross-referencing, with final candidate variant lists and per-sample reports generated using vase_reporter. The resulting somatic variant set was used for tumour purity, ploidy, transition:transversion (Ti/Tv) ratio, mutational signature and sensitivity analyses, while TMB and neoTMB were calculated separately as described below.

### 2.8. TMB Calculation

Tumour mutational burden (TMB) was quantified by calculating the number of non-synonymous, exonic somatic variants per megabase of sequenced DNA (36.6 Mb), restricted to PASS calls supported by ≥4 alt-allele reads with strand-bias evidence for the alt allele on at least one sequencing strand. ‘NeoTMB’ was quantified by excluding variants with a Consequence annotation of exactly “START loss” or “STOP gain” from those used to calculate TMB.

### 2.9. Somatic Mutation Attribution in Multi-Replicate Tumours

In patients with multiple tumour regions, Mutect2 joint calling generates genotype assignments across all analysed tumour samples. To ensure mutation counts reflected evidence present within each individual tumour region, sample-specific mutation attribution was performed using each replicate’s own allele depth and strand-bias support rather than genotype assignment alone. Mutations were attributed to an individual tumour replicate only when supported by at least 4 alt-allele reads with strand-bias evidence for the alt allele on at least one sequencing strand—the same per-sample read-support criteria used in the TMB calculation. This approach was used for mutation count, Ti/Tv ratio, and mutational signature analyses.

### 2.10. Tumour Purity and Ploidy Estimation

Tumour purity and ploidy were estimated for each tumour replicate using PureCN (v2.16.0), which jointly models tumour-versus-normal coverage log-ratios and somatic/germline variant allele fractions to infer the proportion of tumour-derived DNA and the average genomic copy number per sample. Coverage profiles were generated per BAM against the Twist Comprehensive Exome target intervals, GC-normalised, and compared against a pooled reference panel built from all 26 matched normal samples. Base-quality filtering within PureCN (default Phred threshold 25) was disabled (min-base-quality 0), as NovaSeq quality-score binning compresses observed base qualities into a narrow range (Phred 24–29 in this dataset) incompatible with a fixed threshold calibrated for a continuous quality scale; variant-level quality control was otherwise retained via Mutect2′s own statistical filtering. PureCN’s internal quality flags (goodness-of-fit, purity, AT/GC coverage dropout, loss-of-heterozygosity) were retained for each sample and reported alongside purity/ploidy estimates rather than used to exclude samples from primary analysis.

### 2.11. Transition: Transversion Ratio Analysis

The Ti/Tv ratio was calculated per tumour replicate from PASS-filtered somatic single-nucleotide variants meeting the per-replicate read-support criteria described above, using bcftools stats (v1.24). Ti/Tv ratio was evaluated as an indicator of transition enrichment that may occur in the presence of residual cytosine deamination associated with FFPE tissue processing.

### 2.12. Mutational Signature Analysis

To assess the contribution of deamination-associated mutational processes, trinucleotide mutational context profiles (SBS-96) were generated from PASS somatic SNVs using MutationalPatterns and the GRCh38 reference genome. Mutational profiles were decomposed against COSMIC mutational signatures (v3.4) using non-negative least-squares regression. Relative contributions of individual signatures were estimated for each tumour replicate. Attention was paid to SBS1, associated with spontaneous cytosine deamination, and SBS30, which has been reported in association with base-excision repair deficiency and FFPE-related artefacts. Their combined contribution was used as an overall measure of deamination-associated mutagenesis within each sample.

### 2.13. Sensitivity Analysis Evaluation

To evaluate the robustness of cohort-level findings to individual sample characteristics, summary statistics for mutation count, Ti/Tv ratio, and tumour purity were recalculated under four scenarios: (i) the full cohort; (ii) excluding tumour replicates flagged by PureCN for reduced model confidence; (iii) excluding statistical Ti/Tv outliers, defined as values exceeding 1.5× the interquartile range above the third quartile; and (iv) excluding a single sample identified as a mutational outlier on the basis of its mutational signature profile (see Results).

### 2.14. Further Sequencing Analysis

The CoMut plot was visualised in Google Colab python interface using CoMut (version 0.03). Gene lengths were calculated from start and end positions from ENSEMBL using GRCh38 coordinates. The Cancer Genome Atlas Liver Hepatocellular Carcinoma dataset was used to identify previously reported common mutations (https://www.cancer.gov/tcga, accessed 28 February 2025). Pathway analysis was performed using g:Profiler [22]. Phylogenetic trees were created using the Penny branch-and-bound algorithm in PHYLIP (v3.695), applying the Camin–Sokal parsimony method for analysis of binary mutation matrix data.

### 2.15. Immunohistochemistry

FFPE tissue sections were stained with primary antibody as listed in Table 1 or antibody diluent alone as a control (Thermo Fisher) then appropriate horseradish peroxidase (HRP)-conjugated secondary antibody (Vector Laboratories). Positive staining was visualised using ImmPACT DAB HRP Substrate kit (Vector Laboratories). Slides were scanned using either an Aperio GT 450 DX scanner (Leica, Milton Keynes, UK) or AxioScan Z.1 Slidescanner (Zeiss, Oberkochen, Germany). For Haematoxylin and Eosin (H&E) staining, the procedure was the same as above, omitting antibody incubation and DAB visualisation, staining first for haematoxylin, then eosin (both Sigma, Dorset, UK).

### 2.16. Image Analysis

Virtual slides were analysed using QuPath software version 0.5.1 [23]. The positive cell detection feature was used to quantify the percentage of cells positive for Ki-67 staining and the number per millimetre squared of tissue for CD4, CD8 and FoxP3. A small area of each image was used for quality control to achieve ‘positive’ staining as per the datasheet and threshold parameters were adjusted between images if needed. The whole tumour or liver area on the slide was then quantified using the automated ‘positive cell detection’ method. Tumour and background liver regions were annotated using H&E sections and with consultation from a pathologist. Analysis was performed blinded, with scan labels being matched to sample ID after analysis. Exemplar staining for each marker can be seen in Supplementary Figure S1. Where possible, analysis was performed using information from the Leeds Teaching Hospital Trust’s Patient Pathway Manager Plus system to include NHS histopathology reports.

Regional analysis of Ki-67 positivity was performed on tumours where the entire section was captured on the FFPE block. Three concentric circles were created on virtual slides based on the radius of the tumour and the percentage of cells positive for Ki-67 in each circle was measured using QuPath before equations were used to calculate the Ki-67 positivity for each region (Supplementary Figure S2).

### 2.17. Recurrence Analysis

NHS patient records were accessed for dates of surgery and date of recurrence, defined as a radiological or histological diagnosis of recurrent HCC, either intrahepatic or distant metastatic disease. Recurrence-free survival was calculated from the date of surgery to the date of recurrence or last follow-up. For Kaplan–Meier analysis, patients were grouped according to cohort median FoxP3+ T-cell frequency. Univariable Cox proportional hazards was performed using survival and survminer packages in RStudio (v2026.08.02).

### 2.18. Statistics

Statistical analyses were performed using one-way ANOVA, paired *t*-tests, Pearson correlation, two-tailed Spearman rank correlation or Cox proportional hazards regression, as specified in corresponding figure legends. Spearman correlation coefficients are reported with bootstrap 95% confidence intervals calculated using 10,000 resamples. To account for multiple tumours from individual patients, sensitivity analyses were performed using patient-level mean values for multifocal cases. Exploratory two-variable Cox models adjusted the association between FoxP3+ enrichment and recurrence-free survival separately for ordinal TNM stage and tumour number; proportional hazards were assessed using scaled Schoenfeld residuals. Sensitivity analyses excluding patient 52, who met an outlier criterion of 3x IQR for patient-level CD4+ and CD8+ T-cell frequencies, were also performed. Formal study-wide multiplicity correction was not applied because the analyses were exploratory, although Tukey-adjusted *p* values were used for multiple pairwise comparisons following ANOVA. Statistical significance was defined as *p* < 0.05. Analyses and graphs were generated using GraphPad Prism version 10.6.0 and RStudio version 2026.08.2.

## 3. Results

### 3.1. Hepatocellular Carcinoma Tumours Exhibit Wide Genetic Variation

**Figure 1:**
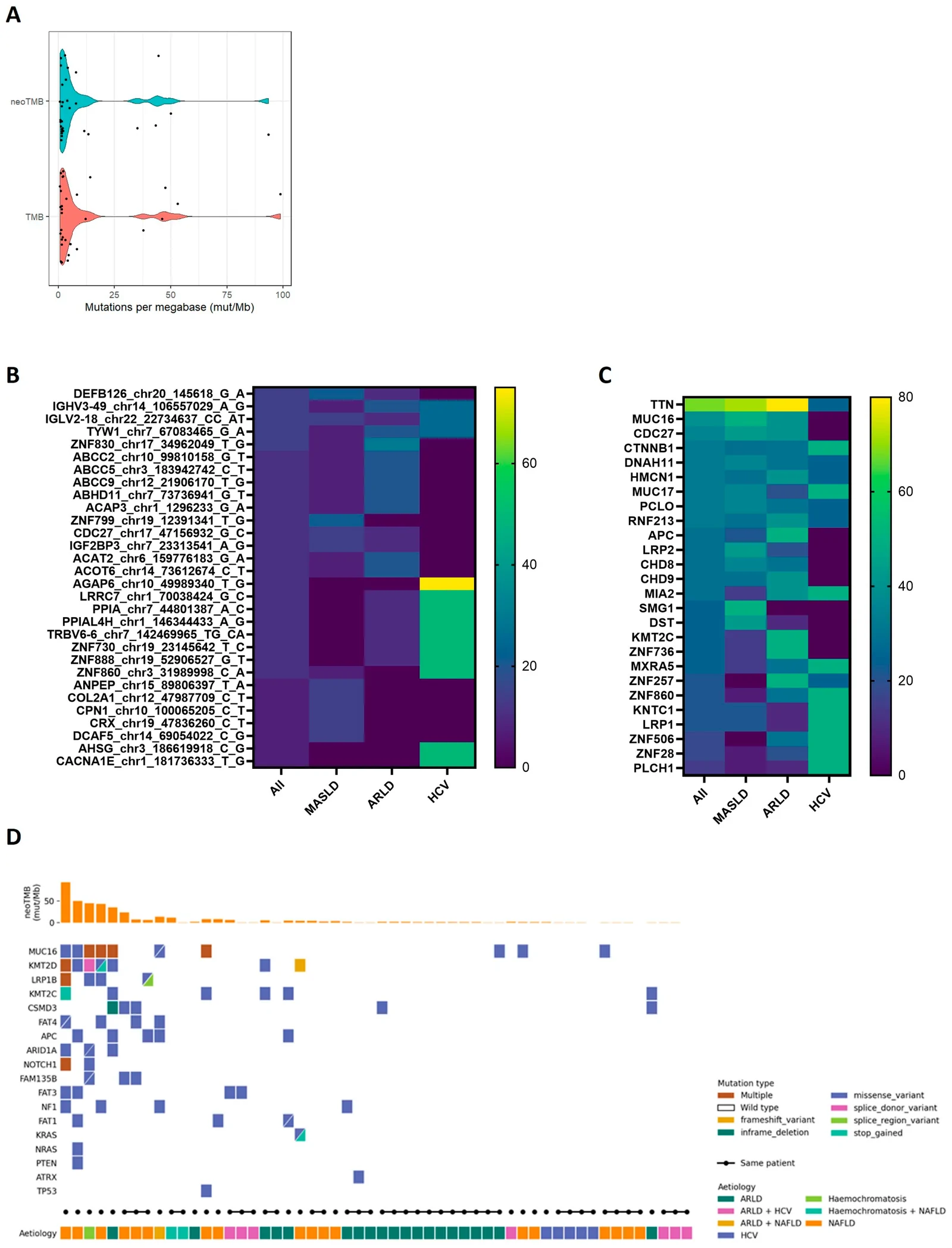
Genetic profiling of HCC tumours overall, by aetiology and the most commonly reported variants. (**A**) Distribution of TMB and neoTMB values. (**B**) Frequency of specific variants found in patients overall and within aetiological groups. Key represents the percentage of tumours that share the variant. (**C**) Frequency of mutated genes found overall and within aetiological groups. Key represents the percentage of tumours that share the mutated gene. (**D**) Occurrence of mutations in hepatocellular carcinoma-associated genes, according to literature reports. Multifocal tumours are indicated by joined circles. For aetiology heatmaps, mutations in multifocal tumours were only counted once per patient. N numbers = 4 to 26.

The burden of tumour-specific somatic variants in this cohort has a median value of 1.44 mutations per megabase (mut/Mb) of sequenced DNA, with significant heterogeneity observed between samples spanning over a 600-fold range (0.16 to 98.63 mut/Mb). Filtering these data for only mutations with the potential to cause neoepitope formation (neoTMB) generated similar results, with a median value of 1.44 mut/Mb and a range of 0.16 to 93.39 mut/Mb (Figure 1A).

Differences in the neoTMB values were not significantly different between aetiological groups, tumour size, gender or differentiation grade (Supplementary Figure S3). However, a significant difference was observed between the neoTMB values for stage T1 tumours when compared to stage T4 tumours (adjusted *p* = 0.03), with a higher neoTMB in T1 tumours (Supplementary Figure S3C). Although not reaching statistical significance, other trends that can be noted are a higher neoTMB in tumours from male patients (median 2.06, range of 0.16 to 93.39) when compared to those from female patients (median 1.41, range of 0.356 to 49.92) and also higher levels in MASLD (median 4.08, range 0.356 to 93.39) when compared to ARLD (median 1.43, range 0.46 to 35.03). No statistically significant association between neoTMB and CD4+ T cells, CD8+ T cells, FoxP3+ T cells or immune cell score was observed (Supplementary Figure S4), and these results were consistent following a patient-level sensitivity analysis using mean values for multifocal cases (Supplementary Table S3). Patient 52, who had received prior radiofrequency ablation and had recorded microvascular invasion, was identified as an extreme patient-level CD4+ and CD8+ T-cell-frequency outlier. Exclusion of this patient did not alter the conclusions of the patient-level sensitivity analyses, with no significant associations identified between neoTMB and either CD4+ or CD8+ T-cell frequency.

To assess the potential impact of FFPE tissue processing on somatic variant detection and tumour mutational burden estimation, additional analyses of tumour purity, ploidy, Ti/Tv ratios, mutational signatures and sensitivity to outlier exclusion were performed.

### 3.2. Tumour Purity and Ploidy

Tumour purity, estimated using PureCN across all 54 tumour replicates, ranged from 0.15 to 0.63 (mean 0.28, median 0.24), consistent with the substantial stromal and immune cell admixture typically observed in FFPE liver tumour biopsies. Estimated ploidy was predominantly near-diploid (median 2.05, range 1.70–3.48), with a minority of samples showing more complex copy-number profiles. Forty-eight of 54 tumour replicates (89%) were flagged by PureCN for reduced confidence in the fitted purity/ploidy solution (reduced goodness-of-fit, low purity, elevated AT/GC coverage dropout, or excessive loss-of-heterozygosity); full purity, ploidy, and flag status for every sample are reported in Supplementary Table S4. Exclusion of flagged samples did not materially alter cohort-level median statistics (see Cohort Sensitivity analysis, below).

### 3.3. Assessment of FFPE Deamination Artefact Contribution to Somatic Calls

To evaluate the extent to which formalin fixation-associated cytosine deamination contributed to called somatic variants, we examined both the Ti/Tv ratio and full COSMIC mutational signature decomposition for each tumour replicate. The cohort median Ti/Tv ratio was 2.33 (mean 4.35, range 0.95–50.63), within a range not indicative of widespread deamination artefacts across the cohort as a whole; the elevated cohort mean reflects a small number of individual samples with skewed ratios, discussed further below. Mutational signature analysis attributed a mean of 7.5% of somatic single-nucleotide variants across the cohort to deamination-associated COSMIC signatures (SBS1 and SBS30 combined), with no sample exceeding 21.7% (Supplementary Table S4). These findings indicate that FFPE-associated deamination artefacts, while detectable at low levels, account for only a small minority of called somatic variants across the cohort and are unlikely to substantially inflate reported tumour mutational burden.

### 3.4. Cohort Sensitivity Analysis

To assess whether cohort-level findings were driven by a small number of individual samples, mutation count, Ti/Tv ratio, and purity were recalculated excluding PureCN-flagged samples, statistical Ti/Tv outliers (1.5× interquartile range above the third quartile), and the mutational outlier described below. Median values for all three metrics remained stable across all exclusion scenarios (Supplementary Table S5), indicating that cohort-level conclusions are not dependent on a small subset of samples.

### 3.5. A Single Tumour Replicate Showed a Mutational Signature Profile Consistent with Mismatch-Repair Deficiency Rather than FFPE Artefacts

One tumour replicate (patient 13, tumour 1) showed a markedly elevated Ti/Tv ratio (50.63, cohort range 0.95–50.63) and the highest somatic mutation count in the cohort (5088 PASS variants, cohort median 163). Given the elevated Ti/Tv ratio, we investigated whether this sample’s mutational profile was consistent with residual FFPE deamination artefacts. Combined SBS1 + SBS30 (deamination-associated) signature contribution in this sample was 12.4%, elevated relative to the cohort mean (7.5%) but not the highest in the cohort (maximum 21.7%, sample 233_1), indicating that deamination artefacts were not the primary driver of this sample’s elevated mutation burden.

Full mutational signature decomposition instead showed dominant contributions from SBS6 (22.7%), a signature associated with defective DNA mismatch repair and microsatellite instability; SBS10b (8.6%), associated with POLE proofreading-domain mutations; and SBS87 (13.3%) and SBS32 (8.4%), both associated with thiopurine/azathioprine exposure. The mutational profile was more compatible with a biological hypermutator phenotype rather than a technical sequencing or fixation artefact, although mismatch-repair deficiency, POLE dysfunction and prior drug exposure were not independently validated. Sensitivity analysis excluding this sample did not materially alter cohort-level median mutation count, Ti/Tv ratio, or purity estimates (Supplementary Table S4), indicating that this sample’s inclusion does not drive the study’s overall conclusions.

### 3.6. Distinct Genetic Patterns Found Between Aetiological Groups

Differing mutational profiles, with respect to individual variants and specific genes containing mutations, are represented in heatmaps in Figure 1B,C. HCV-related tumours shared the highest degree of similarity, with some variants found in at least 50% of tumours, with the same variants occurring rarely in other aetiological groups. Pathway analysis revealed no functional classification of these variants or genes, either overall or with respect to separate aetiological groups (Supplementary Tables S6–S9).

When looking at the cohort of tumours as a whole, there is stark variation in the genes that are mutated. Figure 1D displays a co-mut plot, where individual tumours are represented in columns, showing the most commonly mutated genes according to TCGA project cohorts of liver cancer tumours (*n* = 97). Logically, tumours with a higher neoTMB are seen to contain a higher number of these commonly mutated genes. Tumours from the same aetiological group or same patient do not have a noticeably higher degree of similarity when examining these genes.

Table 2A shows the most frequently mutated genes for all tumours and at an inter-patient level, counting each patient with multifocal tumours once only. The change in frequencies between these measures shows that a high proportion of the shared mutations can be attributed to multifocal patients.

To account for the possibility that gene length might influence mutation frequency, the gene size was used to normalise the inter-patient frequency. The genes with the highest relative frequency are shown in Table 2B. Genes at the top of this include *IGLV2-18* and *TRBV6-5*, which are segments encoding the variable regions of IgG light chains and T-cell receptors, respectively. An ‘inflation’ can be seen where short genes or gene segments are over-represented, and these are smaller in size when compared to the gene lengths in Table 2A (Supplementary Table S10).

### 3.7. Hepatocellular Carcinomas Grow More Rapidly at the Tumour Periphery

**Figure 2:**
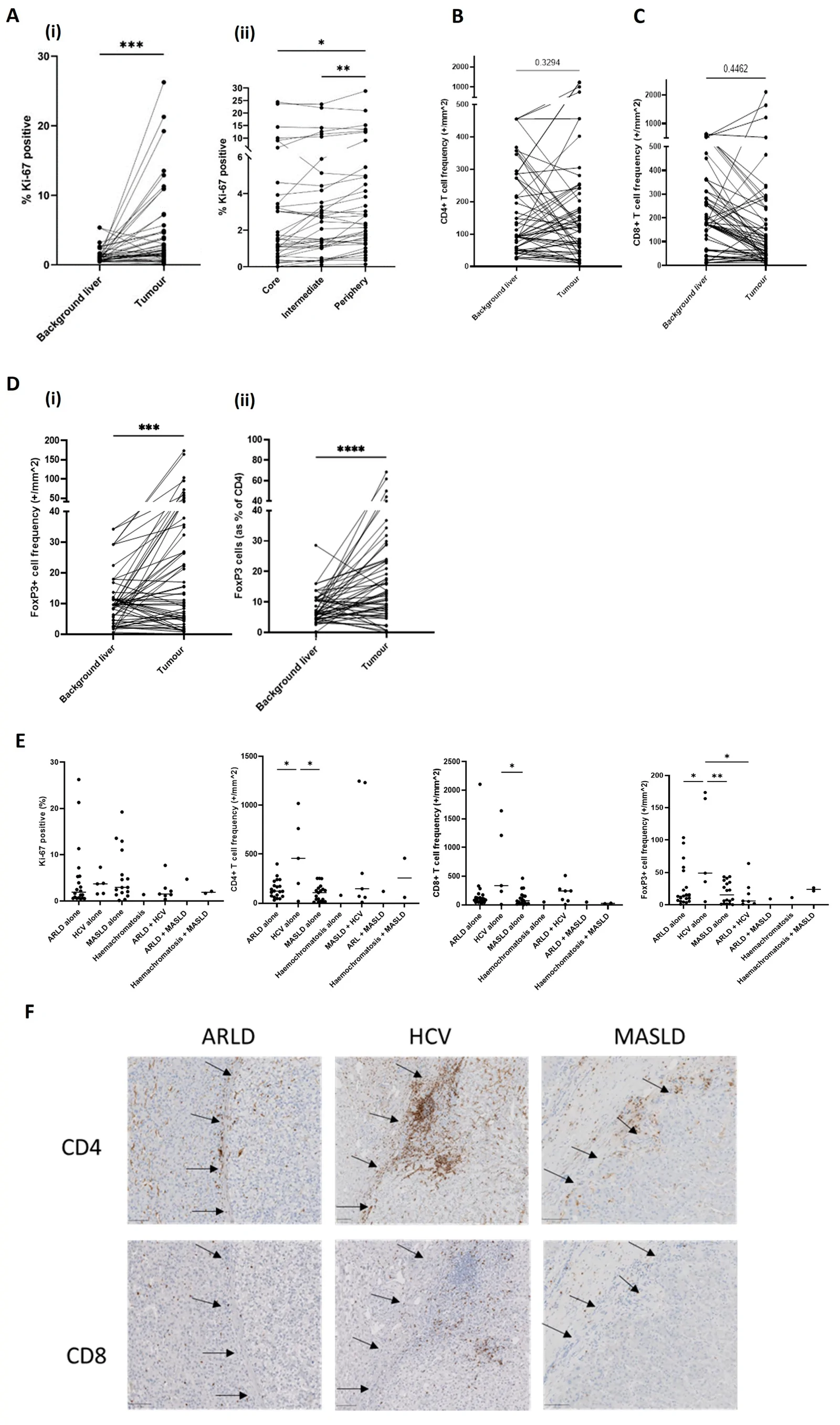
Ki67-positivity and T-cell frequency in paired background liver and HCC samples. (**A**) Ki67-positivity as: (**i**) a percentage of total cells in the paired liver and HCC tissue samples and (**ii**) between concentric regions of the same tumour. The frequency in paired background liver and HCC tissue samples of: (**B**) CD4+ T cells, (**C**) CD8+ T cells and (**D**) (**i**) FoxP3+ T cells and (**ii**) FoxP3+ T cells as a proportion of total CD4+ T cells. (**E**) The frequencies, separated by aetiological group, of Ki-67-positivity, CD4+ T cells, CD8+ T cells and FoxP3+ T cells. (**F**) Representative images of CD4+ and CD8+ T cells at the tumour border (indicated by the arrows) in ARLD, HCV and MASLD patients. Scale bars represent 100 µm. *p* values represent paired *t*-tests (* = *p* < 0.05, ** = *p* < 0.01, *** = *p* < 0.001 and **** = *p* < 0.0001) in sections A to C and One-way ANOVA in sections E to H. N numbers = 30 to 52. The HCV-associated subgroup comprised five tumours from two patients; these observations are therefore exploratory.

Quantification of cellular proliferation, using Ki-67 staining, confirmed that tumours have significantly higher proportions of dividing cells, when compared to paired liver tissue (Figure 2(Ai)). When the entire tumour was captured on the pathology block, analysis of concentric regions revealed a significant increase in the percentage of dividing cells in the tumour periphery when compared to both core and intermediate regions of the same tumour (Figure 2(Aii)).

### 3.8. Enrichment of FoxP3+ but Not of CD4+ or CD8+ T Cells in Tumours

There was no significant increase in the frequency of CD4+ or CD8+ T cells in tumours when compared to sections of background liver from the same patient. There was a large range in the number of tumour-resident cells between tumours, ranging between 8.47 to 1231.86 CD4+ T cells and 3.55 to 1640.43 CD8+ T cells per mm^2^ (Figure 2B,C).

There was, however, a significant increase in the number of FoxP3+ T cells in tumours in comparison to the paired background liver, seen in Figure 2(Di). To account for these cells also being CD4+, the frequency was also plotted as a proportion of CD4+ cells. A greater level of significance was observed in Figure 2(Dii), indicating this increase is independent of the CD4+ frequency in tumours.

No correlation was found between immune cell frequencies and neoTMB (Supplementary Figure S4).

### 3.9. Higher T-Cell Infiltration Was Observed in the HCV-Associated Subgroup

In Figure 2E, Ki-67 quantification shows that proliferation rates were seen to be similar between aetiological groups and that patients with HCV-associated disease had a higher number of tumour-resident CD4+, CD8+ and FoxP3+ T cells when compared to tumours of other aetiological subtypes. This reached significance when compared to the MASLD group for each cell type and also for ARLD in the case of CD4+ and FoxP3+ T cells. This trend is supported by observing the non-significant increase in median value for CD4+ and FoxP3+ cells for the combined ARLD + HCV group. Histology slides revealed aggregation of T cells in HCV-related patient tumours, as seen in comparison to the other aetiological groups in Figure 2F.

### 3.10. Variation in Tumour Proliferation Rate and Frequency of Immune Cells Observed Between Segments of the Liver

**Figure 3:**
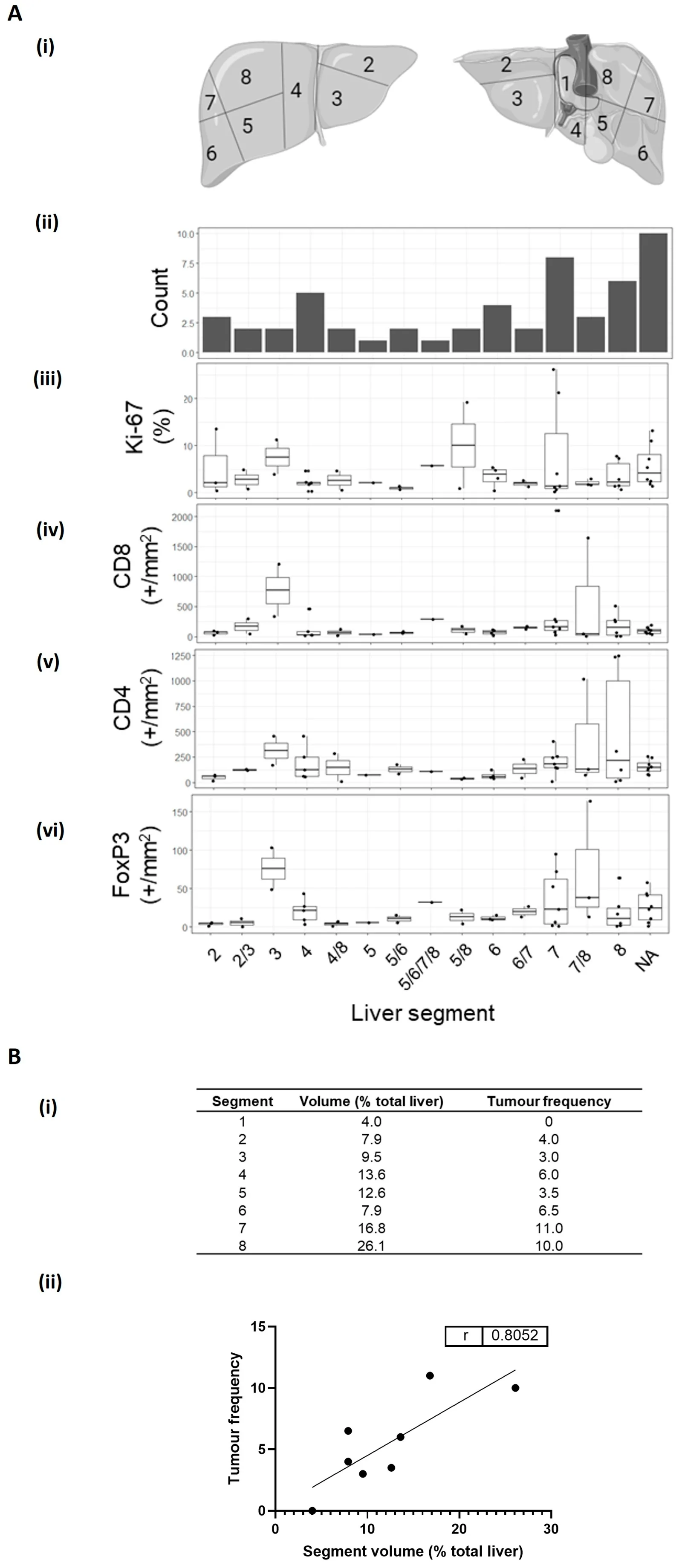
Proliferation, T-cell frequency and tumour occurrence according to segments of the liver. (**A**) (**i**) Diagram of liver segments, (**ii**) segmental location of HCC tumours and frequency of: (**iii**), Ki-67-positivity, (**iv**) tumour-infiltrating CD8+ T cells, (**v**) FoxP3+ T cells and (**vi**) CD4+ T cells by segmental location. Liver segment information was identified from pathology reports by Leeds Teaching Hospitals Trust histopathologists, where available. NA: tumour location not available. N = 51. (**B**) (**i**) Volume of liver segments according to Mise et al. [24] alongside tumour occurrence in each segment and (**ii**) correlation analysis between liver segment volume and tumour frequency. R value indicates the result of a Pearson’s correlation analysis. Created in BioRender. Samson, A. (2026) https://BioRender.com/kebix7n.

Figure 3(Ai) shows the distribution of tumour occurrence by liver segment for those cases where the liver histology was stated on the pathology report. Segments four and seven were the most frequent locations, with no tumours located in segment one. Overall, more tumours were found in the right lobe (segments 4, 5, 6, 7 and 8; 88.7%), when compared to the left lobe (segments 2 and 3; 11.3%).

Although this sample size does not allow for statistical evaluation, data in Figure 3(Aii–vi) show that tumour proliferation and immune cell frequencies vary according to the segment carrying the tumour. Segments three, seven and eight appear to have a trend towards a higher frequency of immune cells when compared to the other segments.

Utilising data from a publication where the average volume of liver segments was modelled from CT scans of 107 normal livers [24] (Figure 3(Bi)), we found a strong correlation between tumour segment location and the average volume of each segment, as seen in Figure 3(Bii).

### 3.11. Multifocal Tumours

**Figure 4:**
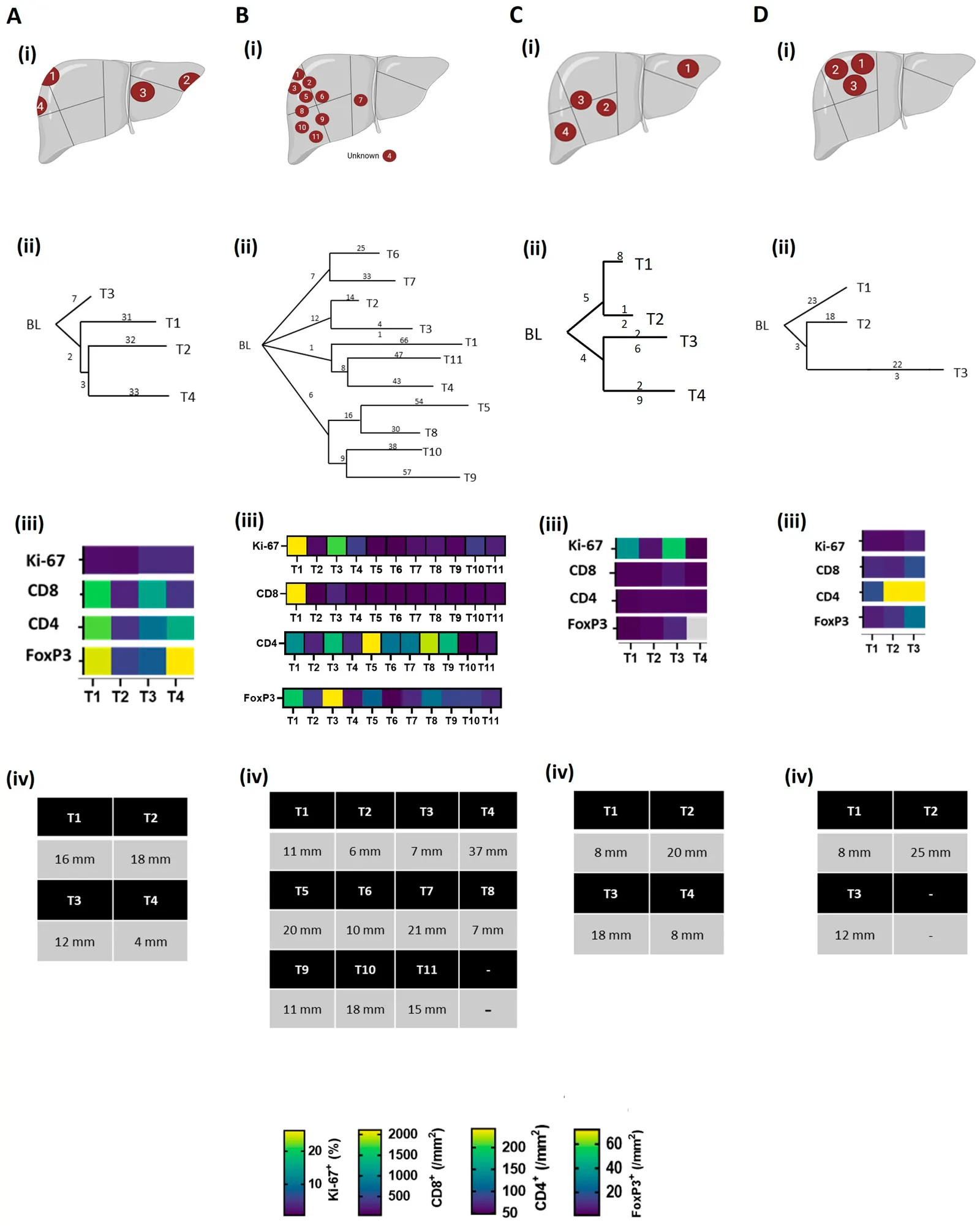
Immunogenetic relatedness of multifocal hepatocellular carcinoma tumours. Data for four multifocal patients (**A**–**D**) to indicate the segmental location of tumours (numbered sequentially) (**i**), genetic similarity according to a Camin–Sokal maximum parsimony phylogenetic tree analysis (**ii**), tumour proliferation and immune cell frequencies (**iii**) and tumour sizes (**iv**). Created in BioRender. Samson, A. (2026) https://BioRender.com/kebix7n. Quantitative pairwise comparisons reported in Supplementary Figure S6.

Of the 10 patients with multifocal tumours, most cases were spread throughout segments of the liver and, in some instances, across the two main lobes. Maximum parsimony analysis to generate phylogenetic trees indicated a range of genetic relatedness contexts, with variants represented by the numbers on dendrogram branches. Often, within the same patients, multifocal tumours showed differing levels of genetic relatedness. Figure 4A shows one patient with four tumours across both main lobes of the liver (i), with three of these having two variants in common whilst tumour (T)3 is completely unrelated (ii). T2 and T4 are the most genetically similar and yet have varied frequencies of FoxP3+ and CD4+ cells (iii) and are remarkably different in size (iv). T1 and T4 are the most similar in immune cell frequencies, except for CD8 T cells, and are located closely within the liver.

A patient with 11 tumours was examined as part of this analysis (Figure 4(Bi)). The phylogenetic tree shows that these tumours fall into four clades and then each tumour contains between 14 and 66 unique variants. Some clades are immunologically similar, such as T6 and T7, which are in neighbouring segments of the liver. Other clades exhibit wide variation in proliferation and immune cell frequency, such as T2 and T3. Tumours in the same clade were found in neighbouring segments for this patient. Figure 4C,D shows another two patients with multifocal tumours, exhibiting variation in tumour location, genetic relatedness and immunohistochemistry characterisation. Diagrams of the other six patients with multifocal tumours can be seen in Supplementary Figure S5. No significant difference in inter-lesional heterogeneity was observed between clonally related and unrelated tumour pairs for any parameter measured, indicating that substantial immune heterogeneity can arise even between genetically related lesions (Supplementary Figure S6).

Next, the similarity of tumours depending on their spatial closeness was evaluated in Supplementary Figure S7. There was no obvious trend for neoTMB (Figure S7A), Ki-67 (Figure S7B), CD4+ T cell (Figure S7C), CD8+ T cell (Figure S7D), FoxP3+ T cell (Figure S7E) or tumour size (Figure S7F). This indicates that the spatial closeness of tumours does not make them more likely to be similar either genetically or with regard to their immune infiltrate.

### 3.12. Relationship to Recurrence

**Figure 5:**
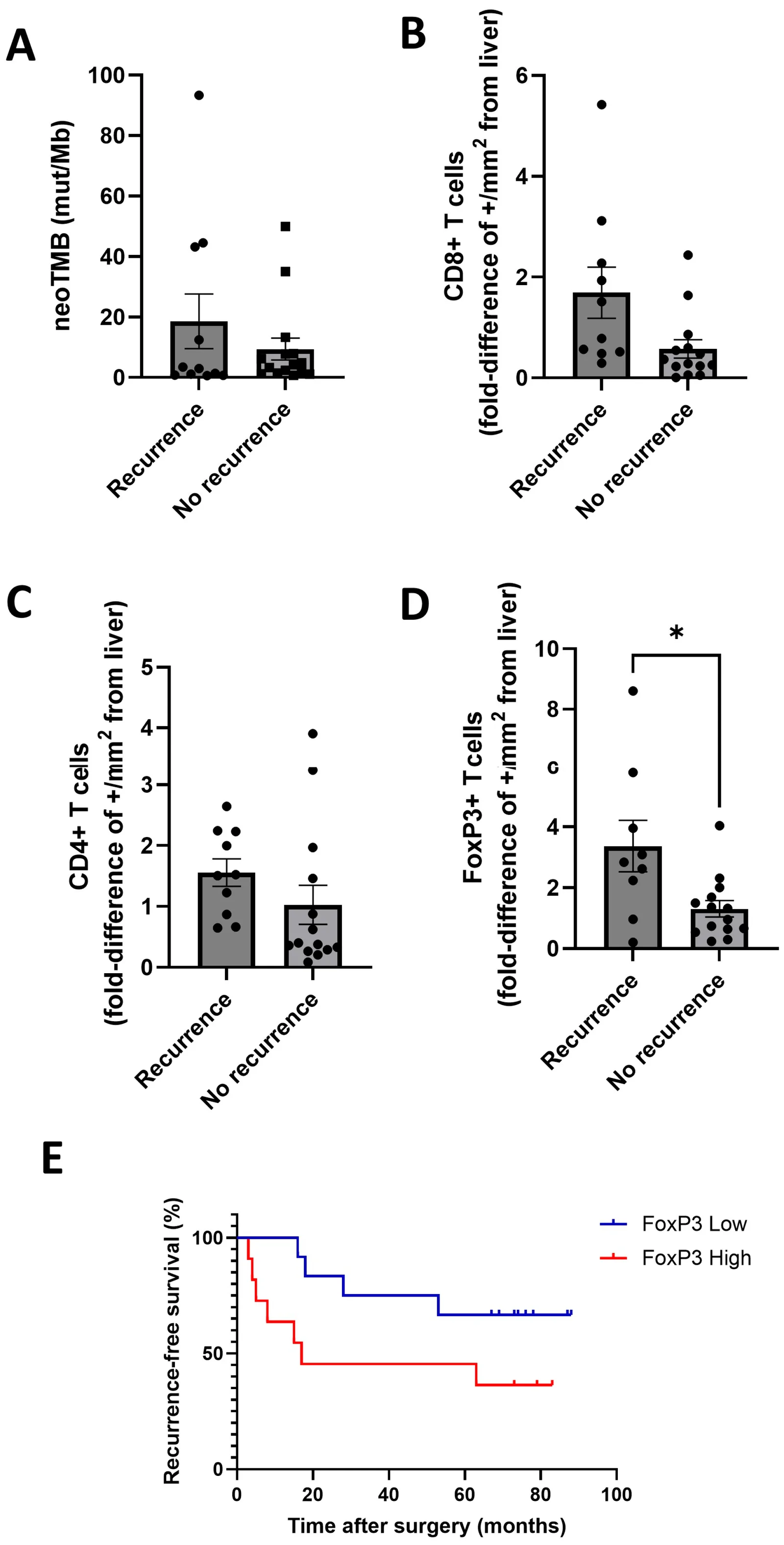
Relationship between neoTMB and immune cell frequencies to recurrence. Bar graphs showing data for each patient based on recurrence status. (**A**) neoTMB values, (**B**) CD8+ T cells, (**C**) CD4+ T cells, (**D**) FoxP3+ T cells. (**E**) Kaplan–Meier plot showing recurrence events, separated into two groups based on cohort median FoxP3 frequency. *p*-value represents unpaired *t*-test (* = *p* < 0.05). Data for multifocal tumours is represented by a mean value.

Following surgical removal of the tumour, the patients included in this study experienced a recurrence rate of 50% after a median follow-up of 6.6 years (range 5.5–12.9 years, Supplementary Table S1). There is no significant difference in neoTMB between patients based on their recurrence status (Figure 5A). For both CD8+ and CD4+ T cells, there is a non-significant trend towards higher frequencies in tumours from patients who experienced a recurrence during the follow-up period (Figure 5B,C). There is however a statistically significant increase in FoxP3+ T-cell frequency in the tumours of patients who presented with HCC recurrence during the follow-up period (*p* = 0.03) (Figure 5D). When the recurrence events were plotted on a Kaplan–Meier plot and separated by ‘high’ or ‘low’ FoxP3+ T-cell frequency, there was a non-significant separation (*p* = 0.08) when using the log-rank test, whereby patients with a high frequency of FoxP3+ T cells had shorter recurrence-free survival (Figure 5E). Univariate Cox proportional hazards regression demonstrated that increasing tumour-to-background FoxP3 enrichment was associated with significantly shorter recurrence-free survival (HR 1.56, 95% CI 1.15–2.13, *p* = 0.0045). TNM stage was also associated with recurrence-free survival (HR per one-stage increase 1.93, 95% CI 1.04 to 3.61, *p* = 0.038), whereas tumour number was not associated with recurrence-free survival (HR per additional tumour 1.02, 95% CI 0.80 to 1.30, *p* = 0.888). In exploratory two-variable models, the association with FoxP3 enrichment was retained after separate adjustment for TNM stage and tumour number. Assessment of Schoenfeld residuals demonstrated no evidence of violation of the proportional hazards assumption, with all global *p* values ≥ 0.61 (Supplementary Table S11). Given the limited number of recurrence events, these analyses should be considered exploratory.

## 4. Discussion

This immunological and genetic study of the main aetiological groups of HCCs within the UK population reflects the high level of heterogeneity between patient tumours and within multifocal disease. The sequencing data generated from these tumours revealed median TMB and neoTMB values both of 1.44 mut/Mb, shown in Figure 1, which is lower than previously reported values in the literature, ranging from 2.45 to 5.4 mut/Mb [6,25,26,27]. Additional analyses of tumour purity, ploidy, mutational signatures and sensitivity to outlier exclusion indicated that deamination-associated mutagenesis contributed only a small proportion of the observed somatic variant burden, supporting the robustness of these findings. However, these data have also highlighted that a considerable range in mutation frequency can occur in tumours both between and within HCC cases. In that respect, our TMB estimations fall within the range of other reports. The finding that T4 tumours have a lower neoTMB than T1 tumours may indicate evolutionary pressures acting during tumour progression, although the mechanisms underlying this observation require further investigation.

Because all sequencing analyses were performed using FFPE-derived tissue, additional analyses were undertaken to assess the potential contribution of fixation-associated artefacts to somatic variant detection. Tumour purity estimates were consistent with the expected stromal and inflammatory admixture observed in liver tumour biopsies, whilst ploidy estimates indicated that most tumours were near-diploid. Importantly, mutational signature analysis demonstrated that deamination-associated signatures (SBS1 and SBS30) contributed a mean of only 7.5% of somatic variants across the cohort, with no sample exceeding 21.7%. Together with the stability of mutation count, tumour purity and Ti/Tv metrics in sensitivity analyses, these findings suggest that deamination-associated mutagenesis accounts for only a minor component of the observed mutational landscape and is unlikely to materially influence the study’s conclusions regarding tumour mutational burden.

The non-significant observation of tumours from male patients having a higher neoTMB does support analysis of TMB from HCC tumours in the TCGA database [28].

Despite substantial variation in neoTMB across the cohort, no correlation was observed with CD4+, CD8+ or FoxP3+ immune cell frequencies, or when represented as an overall T-cell score. These findings are noteworthy considering the widespread interest in TMB as a surrogate marker of immunogenicity. Our data however suggests that the relationship is not straightforward and supports emerging evidence that the immune landscape of HCC is shaped by additional factors. Previous bioinformatic studies using TCGA datasets have found associations between TMB and immune-related transcriptional signatures in HCC, however these analyses frequently infer immune infiltration from gene expression rather than direct quantification of immune cells [28]. By combining whole-exome sequencing with immunohistochemical assessment, our findings provide no evidence that neoTMB is associated with the density of tumour-infiltrating CD4+, CD8+ or FoxP3+ T cells in this exploratory cohort. This conclusion was unchanged in a patient-level sensitivity analysis, although validation in a larger cohort is required.

This observation contrasts with findings in several other tumour types, where TMB has been linked to greater immune cell infiltration and responses to immune checkpoint blockade [29,30]. These differences may reflect the unique immunobiology of HCC, which develops within the chronically inflamed and tolerogenic environment of the liver.

The mutational profiles observed between aetiological groups in Figure 1 allude to different mutagenic processes arising from exposure to distinct risk-factors. For instance, *SMG1* was exclusively found to be mutated in MASLD-associated tumours. This gene encodes an enzyme involved in the nonsense-mediated messenger ribonucleic acid (mRNA) decay process. There is no evident link between the metabolic dysfunction characterising MASLD and SMG1 deregulation; however, the published literature does associate SMG1 haploinsufficiency with tumourigenesis in multiple organs, including in the liver [31]. The overview of commonly mutated genes within this dataset, illustrated by the co-mut plot in Figure 1D, is concordant with previous research regarding these molecular characteristics [32], and also in reporting high inter-tumour variation [33]. Observing a high frequency of mutations in the short gene segments for *IGLV2-18* and *TRBV6-5*, which encode the variable regions of the immunoglobin lambda light chain and T-cell receptor, could point to a concept whereby genes with critical functions are frequently mutated as this is beneficial for tumour evolution, although technical and biological explanations require further investigation.

The gene encoding titin is most frequently mutated between individual patients, at a level of 65.38%. Recent literature describes how mutations in this protein found in liver cancer can increase the protein stability, which has an effect of lowering intracellular ferrous irons levels and thereby suppressing the ferroptosis mechanism of cell death. The same paper also highlights the association between titin mutations and a poor prognosis [34]. Our work corroborates the potential importance of this gene in HCC and justifies its further evaluation.

The immunohistochemistry data presented in Figure 2 evidences the proliferative nature of these tumours and supports a model of boundary-driven growth in HCC, a behaviour common to other cancers [35]. Recently published RNA sequencing data supports the observation presented here [36]. This growth pattern contrasts with examples of selective growth and irregular proliferation at the tumour edge, which occurs in colorectal cancer [37]. One hypothesis is that tumour cells at the periphery have superior access to oxygen and nutrients from the tumour microenvironment (TME) to fuel higher replication rates. This concept is supported by the observation of necrotic cores in some tumours from this dataset. Higher cell replication rates, in turn, accelerate the mutation rate at the periphery, which has also been observed in renal cell carcinoma [38].

A comparison of tumours at the aetiological level revealed that patients with HCV-associated tumours have a significantly higher number of tumour-resident CD4+ and CD8+ T cells, as per Figure 2. This analysis was limited to five tumours found in two patients, and therefore should be confirmed in a larger group of patients; however, the observed higher frequency in tumours from the ARLD + HCV group compared to ARLD alone adds strength to this observation. This data may support findings from some trials indicating that patients with viral aetiologies have a better response to immune checkpoint inhibitor therapy [39], although a meta-analysis shows a significant survival advantage across both viral and non-viral aetiologies when considering data from trials of all immune checkpoint inhibitors in HCC to date [40].

The number of CD4+ or CD8+ T cells in tumours was not higher than those in the background liver from the same patients. This is important when considering that the mechanism of action for current immunotherapy for advanced HCC works to increase the efficacy of an existing immune response. The concept of intrinsic resistance, whereby the therapeutic effect of a treatment is limited before the patient starts treatment [41], could explain why a substantial proportion of patients do not experience tumour shrinkage following atezolizumab–bevacizumab or tremelimumab and durvalumab treatment.

FoxP3+ regulatory T cells have previously been shown to be increased in the HCC TME and were found to be significantly associated with reduced OS in a Chinese HCC cohort [42]. The data presented here also demonstrates a significant increase in the frequency of FoxP3+ T cells in the HCC tumours compared to the background liver in a different patient population, and this is independent of overall CD4+ T-cell frequency. This data evidences the suppressive nature of the HCC tumour immune microenvironment and the potential role of these cells in contributing to immunotherapy resistance.

One unexpected and interesting observation, albeit not sufficiently powered for statistical comparison, is that the frequency of immune cells appears to be influenced by the segment that the tumour is in. A clear trend, shown in Figure 3, is that tumours in segments three, seven and eight have a higher frequency of T cells. The finding of fewer tumours being located in the left lobe is consistent with both this being the smaller of the two main lobes and with the previous literature; a report from a cirrhotic population found that 72.7% of HCC tumours were found in the right lobe [43]. The same study also found that segment one was the least frequent location, which also fits with this data. The strong correlation between segmental volume and tumour occurrence supports a volume-related model of HCC distribution in the liver. Segments of the liver receive differential blood supplies from both branches of the portal vein and the hepatic artery; however, actual segmental blood supply varies widely between patients due to the unpredictable branching of vessels [24]. Despite this, to date, there is no published literature on differences in immune cell frequencies of tumours in different segments. It is reasonable to consider that the vascular accessibility of the tumour to immune cells, dictated by blood flow, plus access to signalling factors, could underlie differences suggested in these data. A study to specifically look at this, with appropriate statistical power, would reveal if this pattern is a true feature of the disease.

With regards to statistical limitations in this study, it is important to note that our cohort has a male bias with a 4.7-fold greater frequency of male patients than females, which is higher than the population-wide male to female ratio of 2.9:1 [44]. As samples were selected based on the most recently FFPE-preserved, to minimise temporal nucleic acid degradation [45], they were not controlled to statistically represent real world data. Surgical and transplant eligibility criteria will have also influenced the demographics of the patients within this dataset; this is determined by the number and size of suspected HCC tumours, hence a skew towards a lower tumour stage is observed. Multifocal tumours are an exception and in some case histopathology findings indicate more tumours were found than suspected from magnetic resonance imaging (MRI) scans. In this set, 35.71% of patients had multifocal disease, which is likely to be an underestimate compared to the population rate due to the confounding effect of surgical and transplant eligibility criteria.

As the samples were collected in an opportunistic manner, as described above, there was no controlling for patient characteristics such as aetiology or tumour node metastasis (TNM) grade. In turn, this means that statistical analyses between groups were not always possible due to insufficient numerical power.

Multiple tumours from the same liver are varied in the frequency of immune cells and the rate of proliferation. This is seen in patients where tumours are demonstrated to share mutations, suggesting metastatic evolution (Figure 4). Published work using RNA sequencing data to infer characteristics of the TME has also noted variation in T-cell infiltration, and between multifocal HCC tumours [20]. Confirming this finding at the proteomic level evidences the extent of intra-patient heterogeneity in multifocal HCC. The presence of multiple tumours with differing frequencies of immune cells is a likely mechanism of intrinsic resistance to the immunotherapy treatments currently used to treat advanced HCC. If there is variation in the immunogenicity of the tumours within the same patient, this is likely to result in a disproportionate anti-tumour response, with some tumours shrinking but others able to continue proliferating, limiting overall treatment efficacy. In this work we see five patients (50%) with tumours that are likely to originate from multiple primaries, and the other half sharing mutations to indicate metastatic origin. Studies in Chinese populations with HBV-associated multifocal HCC have reported a majority of patients having genetically-related tumours [20,21], whereas this work suggests an equal proportion of multiple primary tumours. To our knowledge this is the first publication to characterise multifocal tumours in a wider aetiological cohort and also compare the immune cell frequencies in these patients.

The association between recurrence and FoxP3+ frequency, but not total CD4+ or CD8+ T-cell frequencies, suggests that the immunoregulatory composition of the tumour microenvironment may be more important than overall lymphocyte abundance. This is supported by literature that identified a similar relationship in a cohort of tumours from Chinese patients [46] and also a Japanese study including mostly hepatitis-associated tumours [47]. Although not statistically significant, Kaplan–Meier analysis demonstrated a trend towards shorter recurrence-free survival in patients with higher FoxP3+ T-cell frequencies, supporting the biological relevance of this observation. In addition, the absence of an association between neoTMB and recurrence strengthens the concept that the functional composition of the immune microenvironment has greater clinical relevance than mutational burden alone. Together, these findings suggest that biomarkers reflecting immune suppression may provide superior prognostic information than genomic measures of tumour antigenicity in HCC. The recurrence analysis should be regarded as exploratory because of the limited cohort size and number of recurrence events, precluding a more extensive multivariable model incorporating additional clinicopathological factors. These findings should be regarded as hypothesis-generating and require validation in larger independent cohorts. If this finding were to hold, it could raise the possibility that intratumoural FoxP3+ T-cell frequency, or perhaps looking at a FoxP3:CD8 ratio, may identify patients at high risk of recurrence who could be prioritised for future adjuvant immunotherapy trials. Accounting for further clinical parameters, such as Child–Pugh scores, and validation using fresh-frozen tissue would also strengthen this association.

Given the marked immunological discordance observed between multifocal tumours in this study, assessment of a single lesion may underestimate the presence of immunosuppressive immune phenotypes associated with recurrence. Patients may therefore harbour unsampled lesions with biological characteristics that are more relevant for clinical outcomes than those identified in the sampled tumour. Liquid biopsies offer a minimally invasive approach for characterising circulating tumour DNA (ctDNA) and have shown promise in the diagnosis of HCC [48,49]. By integrating tumour-derived DNA released from multiple lesions, ctDNA analysis may provide a more comprehensive assessment of tumour heterogeneity than conventional single-site tumour biopsy and could aid future risk stratification strategies.

Taken together, the low mutation-driven immunogenicity, poor immune cell infiltration and heterogeneity in multifocal disease support the need for therapeutic approaches that broaden the efficacy of the adaptive anti-tumour immune response. These could include personalised vaccination strategies, oncolytic viruses and strategies for modulating the immunosuppressive TME; all of which could be used in combination with existing standard-of-care immune checkpoint blockades.

## 5. Conclusions

This exploratory study demonstrates substantial genomic and immune heterogeneity between HCCs and among multifocal tumours from the same patient. NeoTMB was not significantly associated with tumour proliferation, T-cell infiltration or recurrence following surgery, and the absence of associations with immune cell frequencies was retained in patient-level sensitivity analyses. In contrast, increased tumour-to-background FoxP3+ T-cell enrichment was associated with shorter recurrence-free survival and this association was retained after separate adjustment for TNM stage and tumour number.

These findings suggest that the immunoregulatory composition of the HCC microenvironment may provide information not captured by mutational burden alone. However, the limited cohort size, small number of recurrence events, heterogeneous aetiologies and absence of external validation mean that the FoxP3 findings should be considered hypothesis-generating. Larger prospective studies incorporating genomic, immune and clinicopathological variables are required.

## Figures and Tables

**Table 1:** Primary antibodies used for immunohistochemistry staining.

Target Antigen	Target Species	Species of Origin	Clone	Dilution	Manufacturer
CD4	Human	Rabbit	Polyclonal	1:100	Abcam
CD8	Human	Mouse	C8/144B	1:100	Agilent
FoxP3	Human	Mouse	236A/E7	1:750	Abcam
Ki-67	Human	Mouse	MIB-1	1:250	Agilent

**Table 2:** The top 10 most commonly mutated genes in this hepatocellular carcinoma cohort. (**A**): Mutation frequency for all tumours and on a per-patient basis. For multifocal patients, mutated genes were just counted once. (**B**): Frequency adjusted for gene length.

**(A)**				
**Gene**	**Frequency**	**Frequency (%)**	**Inter-Patient Frequency**	**Inter-Patient Frequency (%)**
TTN	19	35.19	17	65.38
ZNF708	16	29.63	6	23.08
HMCN1	13	24.07	8	30.77
CDC27	12	22.22	9	34.62
PCLO	12	22.22	8	30.77
ZNF726	12	22.22	7	26.92
DEFB126	11	20.37	4	15.38
ZNF430	11	20.37	3	11.54
ZNF595	11	20.37	5	19.23
MUC16	10	18.52	10	38.46
**(B)**				
**Gene**	**Frequency (%)**	**Gene Size (Mb)**	**Relative Frequency (Frequency/Mb)**	
IGLV2-18	15.38	0.0005	8299	
TRBV6-5	15.38	0.0005	7984	
PPIAL4H	11.54	0.0008	3953	
HERC2	26.92	0.0020	3448	
MT-CYB	11.54	0.0011	2632	
USP17L1	11.54	0.0016	1884	
PRKACG	11.54	0.0016	1865	
RBMXL3	19.23	0.0035	1446	
OLIG3	11.54	0.0022	1367	
ZNF830	11.54	0.0022	1341	

## Data Availability

Whole-exome sequencing raw data has not been uploaded to a repository due to it being identifiable; it can be made available upon reasonable request to the corresponding author.

## Supplementary Materials

The following supporting information can be downloaded at: https://www.mdpi.com/article/10.3390/cancers18193079/s1, Table S1: Characteristics of patient samples; Table S2: Whole-exome sequencing depth and coverage metrics; Table S3: Associations at both tumour and patient levels; Table S4: Whole-exome sequencing purity, ploidy and signatures; Table S5: Sensitivity analysis: Cohort statistics under different exclusion scenarios; Table S6: Pathway analysis of genes mutated in at least 30% of tumours; Table S7: Pathway analysis of genes mutated in at least 30% of ARLD tumours; Table S8: Pathway analysis of genes mutated in at least 30% of all HCV-related tumours; Table S9: Pathway analysis of genes mutated in at least 30% of MASLD-related tumours; Table S10: Gene lengths; Figure S1: Representative IHC images; Figure S2: Concentric image analysis; Figure S3: NeoTMB by patient characteristics; Figure S4: Relationship between neoTMB and Ki-67 or T-cell frequencies; Table S11: Exploratory adjusted Cox regression; Figure S5: Characterisation of multifocal tumours; Figure S6: Quantitative assessment of inter-lesional heterogeneity; Figure S7: Differences according to segmental location.

## References

[1] Sung H., Ferlay J., Siegel R.L., Laversanne M., Soerjomataram I., Jemal A., Bray F. (2021). Global Cancer Statistics 2020: GLOBOCAN Estimates of Incidence and Mortality Worldwide for 36 Cancers in 185 Countries. CA Cancer J. Clin..

[2] Global Burden of Disease Liver Cancer Collaboration (2017). The burden of primary liver cancer and underlying etiologies from 1990 to 2015 at the global, regional, and national level: Results from the global burden of disease study 2015. JAMA Oncol..

[3] Vogel A., Chan S.L., Dawson L.A., Kelley R.K., Llovet J.M., Meyer T., Ricke J., Rimassa L., Sapisochin G., Vilgrain V. (2025). Hepatocellular carcinoma: ESMO Clinical Practice Guideline for diagnosis, treatment and follow-up. Ann. Oncol..

[4] Mauro E., de Castro T., Zeitlhoefler M., Sung M.W., Villanueva A., Mazzaferro V., Llovet J.M. (2025). Hepatocellular carcinoma: Epidemiology, diagnosis and treatment. JHEP Rep..

[5] Chalmers Z.R., Connelly C.F., Fabrizio D., Gay L., Ali S.M., Ennis R., Schrock A., Campbell B., Shlien A., Chmielecki J. (2017). Analysis of 100,000 human cancer genomes reveals the landscape of tumor mutational burden. Genome Med..

[6] Wong M., Kim J.T., Cox B., Larson B.K., Kim S., Waters K.M., Vail E., Guindi M. (2021). Evaluation of tumor mutational burden in small early hepatocellular carcinoma and progressed hepatocellular carcinoma. Hepat. Oncol..

[7] Kim J.Y., Kronbichler A., Eisenhut M., Hong S.H., van der Vliet H.J., Kang J., Shin J.I., Gamerith G. (2019). Tumor mutational burden and efficacy of immune checkpoint inhibitors: A systematic review and meta-analysis. Cancers.

[8] Wu Y., Chen Z., Hu Y., Ren S., Li M.Y. (2026). Rewiring immunometabolism in hepato-biliary-pancreatic cancers: Unraveling TME-mediated metabolic suppression and advancing next-generation immunotherapeutic approaches. Autoimmun. Rev..

[9] Dai Y., Qiang W., Lin K., Gui Y., Lan X., Wang D. (2021). An immune-related gene signature for predicting survival and immunotherapy efficacy in hepatocellular carcinoma. Cancer Immunol. Immunother..

[10] Chen C., Wang Z., Ding Y., Qin Y. (2023). Tumor microenvironment-mediated immune evasion in hepatocellular carcinoma. Front. Immunol..

[11] Ding W., Xu X., Qian Y., Xue W., Wang Y., Du J., Jin L., Tan Y. (2018). Prognostic value of tumor-infiltrating lymphocytes in hepatocellular carcinoma A meta-analysis. Medicine.

[12] Fu J., Xu D., Liu Z., Shi M., Zhao P., Fu B., Zhang Z., Yang H., Zhang H., Zhou C. (2007). Increased Regulatory T Cells Correlate with CD8 T-Cell Impairment and Poor Survival in Hepatocellular Carcinoma Patients. Gastroenterology.

[13] Xagara A., Goulielmaki M., Fortis S.P., Kokkalis A., Chantzara E., Christodoulopoulos G., Samaras I., Saloustros E., Tsapakidis K., Papadopoulos V. (2024). Pre-Existing Immunity Predicts Response to First-Line Immunotherapy in Non-Small Cell Lung Cancer Patients. Cancers.

[14] Ganne-Carrié N., Nahon P. (2025). Differences between hepatocellular carcinoma caused by alcohol and other aetiologies. J. Hepatol..

[15] Llovet J.M., Willoughby C.E., Singal A.G., Greten T.F., Heikenwälder M., El-Serag H.B., Finn R.S., Friedman S.L. (2023). Nonalcoholic steatohepatitis-related hepatocellular carcinoma: Pathogenesis and treatment. Nat. Rev. Gastroenterol. Hepatol..

[16] Diedrich T., Kummer S., Galante A., Drolz A., Schlicker V., Lohse A.W., Kluwe J., Eberhard J.M., Wiesch J.S.Z. (2020). Characterization of the immune cell landscape of patients with NAFLD. PLoS ONE.

[17] Llovet J.M., Castet F., Heikenwalder M., Maini M.K., Mazzaferro V., Pinato D.J., Pikarsky E., Zhu A.X., Finn R.S. (2022). Immunotherapies for hepatocellular carcinoma. Nat. Rev. Clin. Oncol..

[18] Pfister D., Núñez N.G., Pinyol R., Govaere O., Pinter M., Szydlowska M., Gupta R., Qiu M., Deczkowska A., Weiner A. (2021). NASH limits anti-tumour surveillance in immunotherapy-treated HCC. Nature.

[19] Sangro B., Chan S.L., Kelley R.K., Lau G., Kudo M., Sukeepaisarnjaroen W., Yarchoan M., De Toni E.N., Furuse J., Kang Y.K. (2024). Four-year overall survival update from the phase III HIMALAYA study of tremelimumab plus durvalumab in unresectable hepatocellular carcinoma. Ann. Oncol..

[20] Xu L.X., He M.H., Dai Z.H., Yu J., Wang J.G., Li X.C., Jiang B., Ke Z., Su T., Peng Z. (2019). Genomic and transcriptional heterogeneity of multifocal hepatocellular carcinoma. Ann. Oncol..

[21] Xue R., Li R., Guo H., Guo L., Su Z., Ni X., Qi L., Zhang T., Li Q., Zhang Z. (2016). Variable Intra-Tumor Genomic Heterogeneity of Multiple Lesions in Patients with Hepatocellular Carcinoma. Gastroenterology.

[22] Kolberg L., Raudvere U., Kuzmin I., Adler P., Vilo J., Peterson H. (2023). G:Profiler-interoperable web service for functional enrichment analysis and gene identifier mapping (2023 update). Nucleic Acids Res..

[23] Bankhead P., Loughrey M.B., Fernández J.A., Dombrowski Y., McArt D.G., Dunne P.D., McQuaid S., Gray R.T., Murray L.J., Coleman H.G. (2017). QuPath: Open source software for digital pathology image analysis. Sci. Rep..

[24] Mise Y., Satou S., Shindoh J., Conrad C., Aoki T., Hasegawa K., Sugawara Y., Kokudo N. (2014). Three-dimensional volumetry in 107 normal livers reveals clinically relevant inter-segment variation in size. HPB.

[25] Xu Q., Xu H., Deng R., Wang Z., Li N., Qi Z., Zhao J., Huang W. (2021). Multi-omics analysis reveals prognostic value of tumor mutation burden in hepatocellular carcinoma. Cancer Cell Int..

[26] Yin L., Zhou L., Xu R. (2021). Identification of Tumor Mutation Burden and Immune Infiltrates in Hepatocellular Carcinoma Based on Multi-Omics Analysis. Front. Mol. Biosci..

[27] Wang S., Shi H., Liu T., Li M., Zhou S., Qiu X., Wang Z., Hu W., Guo W., Chen X. (2021). Mutation profile and its correlation with clinicopathology in Chinese hepatocellular carcinoma patients. Hepatobiliary Surg. Nutr..

[28] Xie F., Bai Y., Yang X., Long J., Mao J., Lin J., Wang D., Song Y., Xun Z., Huang H. (2020). Comprehensive analysis of tumour mutation burden and the immune microenvironment in hepatocellular carcinoma. Int. Immunopharmacol..

[29] Ricciuti B., Wang X., Alessi J.V., Rizvi H., Mahadevan N.R., Li Y.Y., Polio A., Lindsay J., Umeton R., Sinha R. (2022). Association of High Tumor Mutation Burden in Non-Small Cell Lung Cancers with Increased Immune Infiltration and Improved Clinical Outcomes of PD-L1 Blockade Across PD-L1 Expression Levels. JAMA Oncol..

[30] Marabelle A., Fakih M., Lopez J., Shah M., Shapira-Frommer R., Nakagawa K., Chung H.C., Kindler H.L., Lopez-Martin J.A., Miller W.H. (2020). Association of tumour mutational burden with outcomes in patients with advanced solid tumours treated with pembrolizumab: Prospective biomarker analysis of the multicohort, open-label, phase 2 KEYNOTE-158 study. Lancet Oncol..

[31] Roberts T.L., Ho U., Luff J., Lee C.S., Apte S.H., MacDonald K.P.A., Raggat L.J., Pettit A.R., Morrow C.A., Waters M.J. (2013). Smg1 haploinsufficiency predisposes to tumor formation and inflammation. Proc. Natl. Acad. Sci. USA.

[32] Llovet J.M., Kelley R.K., Villanueva A., Singal A.G., Pikarsky E., Roayaie S., Lencioni R., Koike K., Zucman-Rossi J., Finn R.S. (2021). Hepatocellular carcinoma. Nat. Rev. Dis. Primers.

[33] Rebouissou S., Nault J.C. (2020). Advances in molecular classification and precision oncology in hepatocellular carcinoma. J. Hepatol..

[34] Zhang Z., Sun Y., Zeng Z., Li D., Cao W., Lei S., Chen T. (2025). Identification of the clinical value and biological effects of TTN mutation in liver cancer. Mol. Med. Rep..

[35] Bastola S., Pavlyukov M.S., Yamashita D., Ghosh S., Cho H., Kagaya N., Zhang Z., Minata M., Lee Y., Sadahiro H. (2020). Glioma-initiating cells at tumor edge gain signals from tumor core cells to promote their malignancy. Nat. Commun..

[36] Lewinsohn M.A., Bedford T., Müller N.F., Feder A.F. (2023). State-dependent evolutionary models reveal modes of solid tumour growth. Nat. Ecol. Evol..

[37] Jass J.R., Ajioka Y., Allen J.P., Chan Y.F., Cohen R.J., Nixon J.M., Radojkovic M., Restall A., Stables S., Zwi L. (1996). Assessment of invasive growth pattern and lymphotic infiltration in colorectal cancer. Histopathology.

[38] Hoefflin R., Lahrmann B., Warsow G., Hübschmann D., Spath C., Walter B., Chen X., Hofer L., Macher-Goeppinger S., Tolstov Y. (2016). Spatial niche formation but not malignant progression is a driving force for intratumoural heterogeneity. Nat. Commun..

[39] Haber P.K., Puigvehí M., Castet F., Lourdusamy V., Montal R., Tabrizian P., Buckstein M., Kim E., Villanueva A., Schwartz M. (2021). Evidence-Based Management of Hepatocellular Carcinoma: Systematic Review and Meta-analysis of Randomized Controlled Trials (2002–2020). Gastroenterology.

[40] Meyer T., Galani S., Lopes A., Vogel A. (2023). Aetiology of liver disease and response to immune checkpoint inhibitors: An updated meta-analysis confirms benefit in those with non-viral liver disease. J. Hepatol..

[41] Kelderman S., Schumacher T.N.M., Haanen J.B.A.G. (2014). Acquired and intrinsic resistance in cancer immunotherapy. Mol. Oncol..

[42] Tu J.F., Ding Y.H., Ying X.H., Wu F.Z., Zhou X.M., Zhang D.K., Zou H., Ji J.-S. (2016). Regulatory T cells, especially ICOS+ FOXP3+ regulatory T cells, are increased in the hepatocellular carcinoma microenvironment and predict reduced survival. Sci. Rep..

[43] Renzulli M., Brandi N., Pecorelli A., Pastore L.V., Granito A., Martinese G., Tovoli F., Simonetti M., Dajti E., Colecchia A. (2022). Segmental Distribution of Hepatocellular Carcinoma in Cirrhotic Livers. Diagnostics.

[44] National Health Service, National Disease Registration Service (2025). Cancer Incidence and Mortality. https://nhsd-ndrs.shinyapps.io/incidence_and_mortality/.

[45] Bass B.P., Engel K.B., Greytak S.R., Moore H.M. (2014). A review of preanalytical factors affecting molecular, protein, and morphological analysis of Formalin-Fixed, Paraffin-Embedded (FFPE) tissue: How well do you know your FFPE specimen?. Arch. Pathol. Lab. Med..

[46] Lin S.Z., Chen K.J., Xu Z.Y., Chen H., Zhou L., Xie H.Y., Zheng S.-S. (2013). Prediction of recurrence and survival in hepatocellular carcinoma based on two cox models mainly determined by FoxP3+ regulatory T cells. Cancer Prev. Res..

[47] Kobayashi N., Hiraoka N., Yamagami W., Ojima H., Kanai Y., Kosuge T., Nakajima A., Hirohashi S. (2007). FOXP3+ regulatory T cells affect the development and progression of hepatocarcinogenesis. Clin. Cancer Res..

[48] Sun Y., Wang Q., Zhu P., Li Z., Liu T., Yin R. (2026). Decoding Brain Metastasis with Liquid Biopsy: Biomarkers and Technologies in EGFR-Mutant NSCLC. Cancer Med..

[49] Cui K., Ou Y., Shen Y., Li S., Sun Z. (2020). Clinical value of circulating tumor cells for the diagnosis and prognosis of hepatocellular carcinoma (HCC): A systematic review and meta-analysis. Medicine.

