# Supplementary material for: Tumour Mutation Burden Does Not Predict Immune Cell Frequency or Risk of Recurrence After Surgery in Hepatocellular Carcinoma
Source: Cancers (Basel). 2026 Sep 22;18(19):3079. doi: 10.3390/cancers18193079 (PMC13664604; doi:10.3390/cancers18193079)
Supplement: Supplementary file 1 [file cancers-18-03079-s001.zip › cancers-4554247-supplementary.pdf]

Supplementary Table S1*: **Characteristics of patient samples**, as per NHS pathology reports where available. N/A = no recurrence by last follow-up, RFA = radiofrequency ablation, TACE = transarterial chemoembolization.*

Patient ID	Gender	Aetiology	TNM grade	No. of tumours	Time to recurrence (months)	Tumour size(s, mm)	Cirrhosis	Microvascular invasion	Prior treatment
13	M	MASLD	T2	1	53	25	N	N	NONE
14	M	MASLD	T2	1	63	30	N	N	NONE
25	M	ARLD	T3	3	16	6, 35, 9	Y	N	NONE
44	M	MASLD	T1	1	N/A	7	Y	N	NONE
52	M	HCV	T4	4	8	16, 18, 12, 4	Y	Y	RFA
725	M	ARLD	T2	2	3	15,34	Y	Y	NONE
728	M	ARLD + HCV	T2	3	N/A	8, 25, 12	Y	N	NONE
97	M	ARLD + MASLD	T1	1	N/A	16	Y	N	NONE
10	F	MASLD	T3	1	4	120	N	Y	NONE
17	M	ARLD	T2	1	5	33	Y	Y	NONE
220	M	ARLD	T2	1	N/A	30	Y	N	NONE
221	M	Haemochromatosis	T2	1	28	35	N	N	NONE
222	M	MASLD	T2	2	15	20, 17	N	Y	NONE
223	M	Haemochromatosis + MASLD	T2	2	N/A	8, 6	N	N	TACE
224	F	ARLD	T4	11	N/A	11, 6, 7, 37, 20, 10, 21, 7, 11, 18, 15	Y	Y	NONE
225	M	HCV	T2	1	N/A	25	Y	N	NONE
226	F	MASLD	T4	4	8	8, 20, 18, 8	Y	Y	RFA
227	M	MASLD	T2	1	N/A	25	Y	N	NONE
228	M	ARLD + HCV	T4	4	18	20, 4, 7, 20	Y	N	TACE
229	F	ARLD	T2	1	N/A	35	Y	N	NONE
230	F	MASLD	T1	1	N/A	11	Y	N	NONE
231	F	MASLD	T2	1	N/A	38	Y	N	TACE
232	M	MASLD	T3	3	57	42, 9, 5	N	Y	TACE
233	M	ARLD	T2	1	N/A	27	Y	N	NONE

Supplementary Table S2: Whole exome sequencing depth and coverage metrics

Patient ID	Group	Tumor Replicate	Sample ID	Total Bases	Mean Coverage	Q3 Coverage	Median Coverage	Q1 Coverage	% Bases > 15x
10	normal		10D_S25_L003_trimmed	1,760,848,602	47.0	54	35	22	85.9%
10	tumor	1	10_1D_S26_L003_trimmed	1,445,054,523	38.6	45	28	17	77.7%
13	normal		13D_S1_L003_trimmed	2,769,390,306	74.0	89	71	54	97.8%
13	tumor	1	13_1D_S2_L003_trimmed	1,936,934,357	51.8	68	45	28	90.4%
14	normal		14D_S3_L003_trimmed	3,287,963,619	87.9	105	81	61	98.0%
14	tumor	1	14_1D_S4_L003_trimmed	2,701,647,779	72.2	87	65	48	97.5%
17	normal		17D_S27_L003_trimmed	2,734,941,839	73.1	86	54	35	93.9%
17	tumor	1	17_1D_S28_L003_trimmed	927,473,865	24.8	26	17	10	54.2%
220	normal		220_L011_trimmed	2,907,114,731	77.7	90	61	41	96.6%
220	tumor	1	220_1_L011_trimmed	2,984,663,192	79.7	96	65	43	96.4%
221	normal		221_L011_trimmed	1,666,642,730	44.5	51	32	20	83.9%
221	tumor	1	221_1_L011_trimmed	2,376,001,604	63.5	77	52	35	94.5%
222	normal		222_L011_trimmed	5,985,803,933	159.9	187	143	107	98.3%
222	tumor	1	222_1_L011_trimmed	4,495,057,417	120.1	137	107	82	98.2%
222	tumor	2	222_2_L011_trimmed	3,810,904,863	101.8	121	92	66	97.2%
223	normal		223_L011_trimmed	2,008,168,789	53.7	63	46	32	95.2%
223	tumor	1	223_1_L011_trimmed	2,646,635,797	70.7	89	60	38	94.8%
223	tumor	2	223_2_L011_trimmed	5,298,757,219	141.6	170	128	95	98.0%
224	normal		224_L011_trimmed	4,551,067,564	121.6	159	111	67	96.7%
224	tumor	1	224_1_L011_trimmed	5,439,751,787	145.3	170	135	106	97.9%
224	tumor	10	224_10_L011_trimmed	2,773,563,646	74.1	93	50	19	78.7%
224	tumor	11	224_11_L011_trimmed	4,050,274,187	108.2	128	91	65	97.6%
224	tumor	2a	224_2a_L011_trimmed	2,844,035,509	76.0	102	51	18	77.4%
224	tumor	3a	224_3a_L011_trimmed	2,772,718,600	74.1	100	57	25	83.5%
224	tumor	4	224_4_L011_trimmed	3,467,633,587	92.7	112	79	54	97.0%
224	tumor	5	224_5_L011_trimmed	2,386,680,991	63.8	81	45	17	77.0%
224	tumor	6	224_6_L011_trimmed	2,633,136,537	70.4	87	57	35	93.0%
224	tumor	7	224_7_L011_trimmed	1,760,800,754	47.0	59	33	14	72.2%
224	tumor	8a	224_8a_L011_trimmed	2,272,452,868	60.7	77	45	21	80.8%
224	tumor	9	224_9_L011_trimmed	2,039,145,905	54.5	66	41	23	84.0%
225	normal		225_L011_trimmed	4,146,928,494	110.8	132	99	72	97.7%
225	tumor	1	225_1_L011_trimmed	1,819,292,117	48.6	59	37	23	86.3%
226	normal		226_L011_trimmed	2,679,485,082	71.6	92	47	18	77.7%
226	tumor	1a	226_1a_L011_trimmed	1,956,890,128	52.3	69	42	22	83.5%
226	tumor	2	226_2_L011_trimmed	1,971,072,369	52.7	65	43	27	90.3%
226	tumor	3	226_3_L011_trimmed	2,398,799,056	64.1	81	53	33	92.0%
226	tumor	4	226_4_L011_trimmed	3,065,118,273	81.9	112	66	30	86.6%
227	normal		227_L011_trimmed	3,390,420,061	90.6	104	72	50	97.6%
227	tumor	1a	227_1a_L011_trimmed	1,652,797,394	44.2	59	30	11	68.3%
228	normal		228_L011_trimmed	3,578,885,934	95.6	115	86	62	97.7%
228	tumor	1a	228_1a_L011_trimmed	2,743,147,267	73.3	103	65	33	88.9%
228	tumor	2	228_2_L011_trimmed	4,611,512,553	123.2	159	108	68	96.3%
228	tumor	3	228_3_L011_trimmed	3,952,477,815	105.6	126	96	71	97.9%
228	tumor	4	228_4_L011_trimmed	5,793,099,121	154.8	184	135	98	98.2%
229	normal		229_L011_trimmed	5,141,754,549	137.4	158	120	91	98.1%
229	tumor	1	229_1_L011_trimmed	2,144,648,178	57.3	76	48	25	84.6%
230	normal		230_L011_trimmed	4,695,952,521	125.5	154	118	86	97.8%
230	tumor	1	230_1_L011_trimmed	3,200,015,482	85.5	105	80	59	97.4%
231	normal		231_L011_trimmed	2,434,296,864	65.0	81	60	43	96.3%
231	tumor	1	231_1_L011_trimmed	3,642,512,339	97.3	134	83	47	94.0%
232	normal		232a_L011_trimmed	2,660,296,760	71.1	87	57	36	95.0%
232	tumor	10	232_10_L011_trimmed	3,945,604,662	105.4	129	97	71	97.9%
232	tumor	2	232_2_L011_trimmed	2,017,501,357	53.9	68	47	29	90.2%
232	tumor	3	232_3_L011_trimmed	2,437,720,399	65.1	80	56	37	94.1%
233	normal		233a_L011_trimmed	3,755,932,195	100.4	120	92	68	97.9%
233	tumor	1	233_1_L011_trimmed	2,119,596,444	56.6	74	35	13	71.4%
234	normal		234_pool_L011_trimmed	3,656,356,711	97.7	125	90	60	96.6%
234	tumor	1	234_1_L011_trimmed	6,139,659,161	164.0	202	152	110	98.0%
237	normal		237_pool_L011_trimmed	3,290,698,505	87.9	115	73	43	94.0%
237	tumor	1	237_1_L011_trimmed	1,843,156,225	49.2	62	41	26	90.0%
25	normal		25D_S5_L003_trimmed	4,745,654,946	126.8	151	118	88	98.1%
25	tumor	1	25_1D_S6_L003_trimmed	3,442,035,297	92.0	109	86	65	98.0%
25	tumor	2	25_2D_S7_L003_trimmed	3,656,147,138	97.7	110	83	62	98.0%
25	tumor	3	25_3D_S8_L003_trimmed	2,217,551,296	59.3	70	54	40	97.3%
44	normal		44D_S9_L003_trimmed	1,173,527,840	31.4	39	25	14	72.2%
44	tumor	1	44_1D_S10_L003_trimmed	3,197,002,842	85.4	103	81	61	97.5%
52	normal		52D_S11_L003_trimmed	1,441,983,861	38.5	50	33	20	82.2%
52	tumor	1	52_1D_S12_L003_trimmed	5,702,903,983	152.4	182	144	110	98.1%
52	tumor	2	52_2D_S13_L003_trimmed	4,062,377,316	108.5	131	99	74	97.7%
52	tumor	3	525_3D_S14_L003_trimmed	2,513,208,682	67.2	82	50	30	91.3%
52	tumor	4	52_4D_S15_L003_trimmed	4,051,699,512	108.3	131	103	79	97.7%
725	normal		725D_S16_L003_trimmed	2,096,247,826	56.0	68	52	38	96.1%
725	tumor	1	725_1D_S17_L003_trimmed	1,387,452,646	37.1	44	31	21	87.4%
725	tumor	2	725_2D_S18_L003_trimmed	3,122,194,031	83.4	100	67	44	95.7%
728	normal		728D_S19_L003_trimmed	1,787,902,383	47.8	58	37	23	87.4%
728	tumor	1	728_1D_S20_L003_trimmed	1,870,866,327	50.0	58	38	25	89.4%
728	tumor	2	728_2D_S21_L003_trimmed	3,845,098,529	102.7	122	96	74	97.8%
728	tumor	3	728_3D_S22_L003_trimmed	1,822,923,806	48.7	57	35	21	84.5%
97	normal		97D_1_S23_L003_trimmed	3,352,330,421	89.6	111	76	51	96.2%
97	tumor	1	97_1D_S24_L003_trimmed	4,189,599,390	111.9	130	102	78	97.9%

Supplementary Table S3. Association at both tumour and patient levels.

Associations between neoTMB and Ki-67 positivity or CD8+, CD4+ and FoxP3+ T-cell frequencies were assessed using two-tailed Spearman rank correlation. Tumour-level analyses included all available individual tumour samples. For the patient-level sensitivity analyses, measurements from multifocal tumours were averaged to provide one observation per patient, thereby avoiding the inclusion of multiple observations from the same patient. Ninety-five per cent confidence intervals for Spearman's correlation coefficients were calculated using 10,000 bootstrap resamples. Missing measurements were excluded on a comparison-wise basis.

Footnote: Patient 52, who had received prior radiofrequency ablation and had recorded microvascular invasion, met the 3 × IQR criterion for extreme patient-level CD4+ and CD8+ T-cell frequencies. Sensitivity analyses excluding this patient did not materially alter the results. No significant associations were identified between neoTMB and CD8+ T-cell frequency (Spearman's ρ = -0.084, bootstrap 95% CI -0.503 to 0.343, P = 0.705) or CD4+ T-cell frequency (ρ = -0.033, bootstrap 95% CI -0.440 to 0.396, P = 0.881; N = 23).

Analysis level	Comparison	Spearman's ρ	Bootstrap 95% CI	P value	N
Tumour	neoTMB vs Ki-67	-0.071	-0.338 to 0.217	0.618	51
Tumour	neoTMB vs CD8	-0.121	-0.424 to 0.191	0.396	51
Tumour	neoTMB vs CD4	0.016	-0.291 to 0.324	0.910	51
Tumour	neoTMB vs FoxP3	-0.085	-0.381 to 0.224	0.556	50
Patient	neoTMB vs Ki-67	-0.313	-0.674 to 0.150	0.136	24
Patient	neoTMB vs CD8	-0.181	-0.576 to 0.257	0.396	24
Patient	neoTMB vs CD4	-0.125	-0.525 to 0.314	0.561	24
Patient	neoTMB vs FoxP3	-0.245	-0.631 to 0.227	0.249	24

Supplementary Table S4: Whole Exome sequencing purity, ploidy and signatures.

Patient ID	Tumor Replicate	Sample ID	PASS Mutations	Transitions	Transversions	Ti/Tv Ratio	Purity	Ploidy	PureCN Flagged	PureCN Comment	SBS1 Contribution	SBS30 Contribution	Deamination Signature Total (SBS1+SBS30)	Mean Coverage	% Bases > 15x
10	1	10_1	422	293	106	2.76	0.60	2.11	TRUE	POOR GOF (65.4%);HIGH AT- OR GC-DRO	13.1%	8.6%	21.7%	38.6	77.7%
13	1	13_1	5088	4658	92	50.63	0.51	1.99	TRUE	POOR GOF (48.5%)	5.5%	6.9%	12.4%	51.8	90.4%
14	1	14_1	1368	1169	187	6.25	0.47	2.10	TRUE	POOR GOF (76.3%)	2.4%	12.2%	14.6%	72.2	97.5%
17	1	17_1	342	208	114	1.82	0.30	2.63	TRUE	POOR GOF (73%);HIGH AT- OR GC-DROP	11.2%	3.1%	14.3%	24.8	54.2%
220	1	220_1	1731	1540	119	12.94	0.47	2.09	TRUE	POOR GOF (79.9%)	3.0%	14.0%	17.0%	79.7	96.4%
221	1	221_1	2451	2010	281	7.15	0.15	2.04	TRUE	LOW PURITY	4.7%	4.8%	9.4%	63.5	94.5%
222	1	222_1	125	58	49	1.18	0.38	1.98	FALSE	NA	7.3%	1.1%	8.4%	120.1	98.2%
222	2	222_2	99	52	37	1.41	0.15	1.87	TRUE	LOW PURITY;EXCESSIVE LOH	11.6%	0.0%	11.6%	101.8	97.2%
223	1	223_1	133	92	33	2.79	0.32	2.05	TRUE	POOR GOF (74.4%)	7.9%	0.0%	7.9%	70.7	94.8%
223	2	223_2	316	279	27	10.33	0.16	2.04	TRUE	LOW PURITY	9.3%	5.7%	15.1%	141.6	98.0%
224	10	224_10	121	67	39	1.72	0.54	2.20	TRUE	POOR GOF (71.4%)	1.9%	0.0%	1.9%	74.1	78.7%
224	11	224_11	146	84	54	1.56	0.20	1.70	TRUE	LOW PURITY;EXCESSIVE LOH	0.0%	0.0%	0.0%	108.2	97.6%
224	1	224_1	183	127	42	3.02	0.23	1.96	TRUE	LOW PURITY	0.0%	0.0%	0.0%	145.3	97.9%
224	2	224_2	50	32	14	2.29	0.15	2.08	TRUE	LOW PURITY	3.6%	0.0%	3.6%	76.0	77.4%
224	3	224_3	121	69	37	1.86	0.32	2.04	TRUE	POOR GOF (72%)	0.1%	0.0%	0.1%	74.1	83.5%
224	4	224_4	140	78	46	1.70	0.44	3.48	FALSE	NA	4.1%	0.0%	4.1%	92.7	97.0%
224	5	224_5	143	81	43	1.88	0.39	2.11	TRUE	POOR GOF (71.8%)	0.0%	0.0%	0.0%	63.8	77.0%
224	6	224_6	76	41	28	1.46	0.15	2.18	TRUE	LOW PURITY;EXCESSIVE LOH	3.1%	0.0%	3.1%	70.4	93.0%
224	7	224_7	126	64	41	1.56	0.28	2.12	TRUE	LOW PURITY;POOR GOF (69.6%)	0.4%	0.0%	0.4%	47.0	72.2%
224	8	224_8	138	80	40	2.00	0.26	2.01	TRUE	NON-ABERRANT;LOW PURITY;POOR GOI	0.4%	0.0%	0.4%	60.7	80.8%
224	9	224_9	271	154	82	1.88	0.33	2.06	TRUE	POOR GOF (68.3%)	1.9%	0.0%	1.9%	54.5	84.0%
225	1	225_1	259	158	77	2.05	0.36	1.84	TRUE	POOR GOF (69.8%)	6.0%	9.1%	15.0%	48.6	86.3%
226	1	226_1	147	103	39	2.64	0.23	2.02	TRUE	LOW PURITY;POOR GOF (71%)	0.0%	7.5%	7.5%	52.3	83.5%
226	2	226_2	126	87	31	2.81	0.19	1.98	TRUE	LOW PURITY;POOR GOF (73.1%)	4.0%	0.0%	4.0%	52.7	90.3%
226	3	226_3	332	238	83	2.87	0.19	2.13	TRUE	LOW PURITY;POOR GOF (78.4%)	0.0%	13.5%	13.5%	64.1	92.0%
226	4	226_4	159	111	36	3.08	0.21	1.95	TRUE	LOW PURITY;POOR GOF (74.7%)	2.5%	10.3%	12.8%	81.9	86.6%
227	1	227_1	337	241	70	3.44	0.15	2.19	TRUE	LOW PURITY;EXCESSIVE LOH;HIGH AT- (	6.2%	2.8%	9.0%	44.2	68.3%
228	1	228_1	14	6	6	1.00	0.27	1.99	TRUE	LOW PURITY;POOR GOF (74.4%)	0.0%	0.0%	0.0%	73.3	88.9%
228	2	228_2	21	15	5	3.00	0.38	1.99	FALSE	NA	0.0%	2.2%	2.2%	123.2	96.3%
228	3	228_3	41	32	9	3.56	0.18	2.01	TRUE	LOW PURITY	0.0%	0.0%	0.0%	105.6	97.9%
228	4	228_4	125	64	55	1.16	0.57	2.09	TRUE	HIGH AT- OR GC-DROPOUT	0.1%	0.7%	0.8%	154.8	98.2%
229	1	229_1	328	200	119	1.68	0.17	1.99	TRUE	LOW PURITY;POOR GOF (77%)	0.9%	12.7%	13.6%	57.3	84.6%
230	1	230_1	2717	2504	146	17.15	0.15	2.09	TRUE	LOW PURITY	1.5%	15.6%	17.1%	85.5	97.4%
231	1	231_1	564	469	67	7.00	0.44	2.31	TRUE	POOR GOF (78.8%)	0.0%	0.0%	0.0%	97.3	94.0%
232	10	232_10	771	659	88	7.49	0.15	2.04	TRUE	LOW PURITY	3.2%	18.4%	21.6%	105.4	97.9%
232	2	232_2	617	508	50	10.16	0.15	2.06	TRUE	LOW PURITY;POOR GOF (77.7%);EXCESS	5.0%	15.8%	20.8%	53.9	90.2%
232	3	232_3	447	330	51	6.47	0.15	2.07	TRUE	LOW PURITY;EXCESSIVE LOH	0.8%	0.8%	1.6%	65.1	94.1%
233	1	233_1	209	126	60	2.10	0.15	2.26	TRUE	LOW PURITY;EXCESSIVE LOH;HIGH AT- (	18.3%	3.4%	21.7%	56.6	71.4%
234	1	234_1	218	100	105	0.95	0.39	3.17	TRUE	HIGH AT- OR GC-DROPOUT	4.4%	6.6%	11.0%	164.0	98.0%
237	1	237_1	435	273	125	2.18	0.63	2.06	TRUE	POOR GOF (66.2%)	0.0%	2.0%	2.0%	49.2	90.0%
25	1	25_1	35	26	8	3.25	0.15	2.03	TRUE	LOW PURITY;EXCESSIVE LOH	0.3%	5.9%	6.2%	92.0	98.0%
25	2	25_2	168	99	64	1.55	0.41	2.07	FALSE	NA	4.7%	2.7%	7.4%	97.7	98.0%
25	3	25_3	24	14	8	1.75	0.15	1.98	TRUE	LOW PURITY;EXCESSIVE LOH	0.0%	0.0%	0.0%	59.3	97.3%
44	1	44_1	370	324	42	7.71	0.15	1.95	TRUE	LOW PURITY;EXCESSIVE LOH	0.8%	1.3%	2.1%	85.4	97.5%
52	3	52_3	28	17	11	1.55	0.15	2.17	TRUE	LOW PURITY	0.0%	0.0%	0.0%	67.2	91.3%
52	1	52_1	83	42	33	1.27	0.15	2.10	TRUE	LOW PURITY	1.8%	0.0%	1.8%	152.4	98.1%
52	2	52_2	129	82	41	2.00	0.30	2.09	FALSE	NA	0.0%	2.8%	2.8%	108.5	97.7%
52	4	52_4	58	33	19	1.74	0.15	1.99	TRUE	LOW PURITY;HIGH AT- OR GC-DROPOUT	8.9%	0.0%	8.9%	108.3	97.7%
725	1	725_1	167	115	47	2.45	0.30	2.03	TRUE	POOR GOF (68.6%);HIGH AT- OR GC-DRO	10.3%	0.0%	10.3%	37.1	87.4%
725	2	725_2	268	188	59	3.19	0.25	2.04	TRUE	LOW PURITY	6.4%	0.0%	6.4%	83.4	95.7%
728	1	728_1	83	58	21	2.76	0.18	2.01	TRUE	NON-ABERRANT;LOW PURITY;POOR GOI	10.9%	2.9%	13.9%	50.0	89.4%
728	2	728_2	275	194	72	2.69	0.15	2.35	TRUE	LOW PURITY;HIGH AT- OR GC-DROPOUT	9.6%	5.0%	14.5%	102.7	97.8%
728	3	728_3	72	37	25	1.48	0.30	2.00	TRUE	NON-ABERRANT;POOR GOF (71.7%)	2.4%	0.0%	2.4%	48.7	84.5%
97	1	97_1	321	223	94	2.37	0.30	2.03	FALSE	NA	2.5%	3.4%	6.0%	111.9	97.9%

Supplementary Table S5: Sensitivity Analysis: Cohort Statistics Under Different Exclusion Scenarios

Scenario	n	Median Mutation Count	Mean Mutation Count	Median Ti/Tv Ratio	Mean Ti/Tv Ratio	Median Purity	Mean Purity
Full cohort	54	163.0	435.9	2.3	4.4	0.24	0.28
Excluding PureCN-flagged samples	6	134.5	150.7	1.9	2.0	0.38	0.37
Excluding Ti/Tv statistical outliers (>5.6, 1.5×IQR)	43	138.0	165.1	2.0	2.1	0.26	0.28
Excluding documented hypermutator (13_1)	53	159.0	348.1	2.3	3.5	0.23	0.27
**Samples excluded under Ti/Tv outlier criterion (n=11):**							
13_1, 14_1, 220_1, 221_1, 223_2, 230_1, 231_1, 232_2, 232_3, 232_10, 44_1							
**PureCN-flagged samples excluded (n=48):**							
See Supplementary Table S3, 'PureCN Flagged' column, for the full per-sample flag status and reason.							

source	term_name	term_id	adjusted_p_value	negative_log10_of_adjusted_p_value	term_size	query_size	intersection_size	effective_domain_size	intersections
GO:BP	detection of muscle stretch	GO:0035995	0.003796073	2.420665443	7	9	2	21026	TTN,CTNNB1
GO:BP	synaptic vesicle clustering	GO:0097091	0.021648547	1.664571247	16	9	2	21026	CTNNB1,PCLO
GO:CC	presynaptic active zone cytoplasmic component	GO:0098831	0.002749454	2.560753538	18	9	2	22149	CTNNB1,PCLO
GO:CC	cell cortex region	GO:0099738	0.01127841	1.947752104	36	9	2	22149	CTNNB1,PCLO
GO:CC	cytoplasmic region	GO:0099568	0.027080454	1.567344062	313	9	3	22149	CTNNB1,DNAH11,PCLO
GO:CC	extracellular region	GO:0005576	0.029610294	1.52855728	4258	9	7	22149	TTN,MUC16,CTNNB1,MUC17,HMCN1,DNAH11,PCLO
GO:CC	cell cortex	GO:0005938	0.031081539	1.50749748	328	9	3	22149	CTNNB1,HMCN1,PCLO
GO:CC	proximal portion of axoneme	GO:0120134	0.049918782	1.301736019	1	9	1	22149	DNAH11
REAC	Defective GALNT12 causes CRCS1	REAC:R-HSA-5083636	0.009550725	2.019963656	16	7	2	11004	MUC16,MUC17
REAC	Defective GALNT3 causes HFTC	REAC:R-HSA-5083625	0.009550725	2.019963656	16	7	2	11004	MUC16,MUC17
REAC	Defective C1GALT1C1 causes TNPS	REAC:R-HSA-5083632	0.010820874	1.96573765	17	7	2	11004	MUC16,MUC17
REAC	Termination of O-glycan biosynthesis	REAC:R-HSA-977068	0.020093424	1.696946057	23	7	2	11004	MUC16,MUC17
REAC	Dectin-2 family	REAC:R-HSA-5621480	0.025788241	1.588578281	26	7	2	11004	MUC16,MUC17
CORUM	TCF4-CTNNB1 complex	CORUM:5262	0.049931693	1.301623705	1	2	1	3383	CTNNB1
CORUM	Cell-cell junction complex (CDH1-CTNNB1)	CORUM:5281	0.049931693	1.301623705	1	2	1	3383	CTNNB1
CORUM	CTNNB1-DNMT1 complex	CORUM:6336	0.049931693	1.301623705	1	2	1	3383	CTNNB1

Supplementary Table S6: *Pathway analysis of genes mutated in at least 30% of all tumours; patients with multifocal disease are only represented once.*

Supplementary Table S7: *Pathway analysis of genes mutated in at least 30% of all ARLD tumours; patients with multifocal disease are only represented once.*

term_name	term_id
ion binding	GO:0043167
RNA polymerase II cis-regulatory region sequence-specific DNA binding	GO:0000978
cis-regulatory region sequence-specific DNA binding	GO:0000987
small molecule binding	GO:0036094
RNA polymerase II transcription regulatory region sequence-specific DNA binding	GO:0000977
cation binding	GO:0043169
metal ion binding	GO:0046872
double-stranded DNA binding	GO:0003690
transcription cis-regulatory region binding	GO:0000976
transcription regulatory region nucleic acid binding	GO:0001067
DNA-binding transcription factor activity	GO:0003700
sequence-specific double-stranded DNA binding	GO:1990837
microtubule motor activity	GO:0003777
sequence-specific DNA binding	GO:0043565
DNA binding	GO:0003677
DNA-binding transcription factor activity, RNA polymerase II-specific	GO:0000981
transcription regulator activity	GO:0140110
minus-end-directed microtubule motor activity	GO:0008569
cytoskeletal motor activity	GO:0003774
polypeptide conformation or assembly isomerase activity	GO:0120544
dynein light intermediate chain binding	GO:0051959
macromolecular conformation isomerase activity	GO:0120543
regulation of cellular process	GO:0050794
regulation of biological process	GO:0050789
regulation of transcription by RNA polymerase II	GO:0006357
transcription by RNA polymerase II	GO:0006366
regulation of primary metabolic process	GO:0080090
DNA-templated transcription	GO:0006351
biological regulation	GO:0065007
regulation of DNA-templated transcription	GO:0006355
regulation of RNA biosynthetic process	GO:2001141
supramolecular polymer	GO:0099081
supramolecular fiber	GO:0099512
Z disc	GO:0030018
supramolecular complex	GO:0099080
I band	GO:0031674
cytoskeleton	GO:0005856
microtubule	GO:0005874
microtubule cytoskeleton	GO:0015630
microtubule associated complex	GO:0005875
sarcomere	GO:0030017
Herpes simplex virus 1 infection	KEGG:05168
Factor: PBX3; motif: NNNTGAGTGACAGGNNN	TF:M09781
Factor: NF-Y; motif: NCTGATTGGYTASY; match class: 1	TF:M00209_1
Factor: NFYA; motif: RRCCAATCAGN; match class: 1	TF:M09816_1
Factor: PREP-1; motif: TGANTGRCAGS; match class: 1	TF:M10032_1
Factor: PKNOX1; motif: NNTGAGTGACAGNNN	TF:M12559
hsa-mir-4504	MIRNA:hsa-mir-4504
hsa-mir-4768-5p	MIRNA:hsa-mir-4768-5p
hsa-mir-548an	MIRNA:hsa-mir-548an
hsa-mir-549a	MIRNA:hsa-mir-549a
hsa-mir-4484	MIRNA:hsa-mir-4484
hsa-mir-3674	MIRNA:hsa-mir-3674

source	term_name	term_id	adjusted_p_value	negative_log10_of_adjusted_p_value	term_size	query_size	intersection_size	effective_domain_size	intersections
GO:BP	lipoprotein transport	GO:0042953	0.044773557	1.348978403	20	10	2	21026	MIA2,LRP1
GO:BP	lipoprotein localization	GO:0044872	0.049474008	1.305622902	21	10	2	21026	MIA2,LRP1
WP	Regulation of Wnt B catenin signaling by small molecule compounds	WP:WP3664	0.000694656	3.15823022	17	3	2	8752	CTNNB1,LRP1
CORUM	TCF4-CTNNB1 complex	CORUM:5262	0.049987986	1.301134359	1	3	1	3383	CTNNB1
CORUM	Cell-cell junction complex (CDH1-CTNNB1)	CORUM:5281	0.049987986	1.301134359	1	3	1	3383	CTNNB1
CORUM	CTNNB1-DNMT1 complex	CORUM:6336	0.049987986	1.301134359	1	3	1	3383	CTNNB1
CORUM	CD44-LRP1 complex	CORUM:7535	0.049987986	1.301134359	1	3	1	3383	LRP1

Supplementary Table S8: *Pathway analysis of genes mutated in at least 30% of all HCV-related tumours; patients with multifocal disease are only represented once.*

term_id	adjusted_p_value	negative_log10_of_adjusted_p_value	term_size	query_size	intersection_size	effective_domain_size	intersections
GO:0008569	0.000681778	3.166356817	17	29	3	20246	DNAH11,DNAH1,DYNC1H1
GO:0051959	0.003249947	2.488123763	28	29	3	20246	DNAH11,DNAH1,DYNC1H1
GO:0051015	0.00417151	2.379706715	209	29	5	20246	TTN,SPTBN4,MPRIP,SPTAN1,SYNE1
GO:0005198	0.005979805	2.223312977	1106	29	9	20246	TTN,DST,MUC17,PCLO,SPTBN4,EPPK1,SPTAN1,AHNAK,LAMA2
GO:0045505	0.007641685	2.116810895	37	29	3	20246	DNAH11,DNAH1,DYNC1H1
GO:0003779	0.012483681	1.903657326	442	29	6	20246	TTN,DST,SPTBN4,MPRIP,SPTAN1,SYNE1
GO:0003777	0.047839363	1.320214616	68	29	3	20246	DNAH11,DNAH1,DYNC1H1
GO:0097493	0.049843398	1.302392362	12	29	2	20246	TTN,AHNAK
GO:0006996	0.005617786	2.250434813	3594	26	15	21026	TTN,SMG1,CDC27,DST,PCLO,SPTBN4,DNAH1,DYNC1H1,EPPK1,GCN1,MKI67,SPTAN1,ESPL1,SYNE1,UBR4
GO:0005856	9.54E-07	6.020349571	2474	28	16	22149	TTN,CDC27,DST,DNAH11,PCLO,SPTBN4,MPRIP,DNAH1,DYNC1H1,EPPK1,SPTAN1,ESPL1,HERC2,SYNE1,AHNAK,UBR4
GO:0015629	0.00072638	3.138835938	534	28	7	22149	TTN,DST,SPTBN4,MPRIP,SPTAN1,SYNE1,AHNAK
GO:0099512	0.000857404	3.066814339	1068	28	9	22149	TTN,DST,DNAH11,SPTBN4,DNAH1,DYNC1H1,EPPK1,SYNE1,AHNAK
GO:0099081	0.000911348	3.040315948	1076	28	9	22149	TTN,DST,DNAH11,SPTBN4,DNAH1,DYNC1H1,EPPK1,SYNE1,AHNAK
GO:0099080	0.001277722	2.893563792	1448	28	10	22149	TTN,SMG1,DST,DNAH11,SPTBN4,DNAH1,DYNC1H1,EPPK1,SYNE1,AHNAK
GO:0043232	0.00162891	2.788102909	6669	28	20	22149	TTN,SMG1,CDC27,DST,DNAH11,PCLO,SPTBN4,CHD8,MPRIP,DNAH1,DYNC1H1,EPPK1,GCN1,MKI67,SPTAN1,ESPL1,HERC2,SYNE1,AHNAK,UBR4
GO:0043228	0.001633029	2.787006176	6670	28	20	22149	TTN,SMG1,CDC27,DST,DNAH11,PCLO,SPTBN4,CHD8,MPRIP,DNAH1,DYNC1H1,EPPK1,GCN1,MKI67,SPTAN1,ESPL1,HERC2,SYNE1,AHNAK,UBR4
GO:0042995	0.003645582	2.438233176	2441	28	12	22149	LRP2,DST,DNAH11,PCLO,SPTBN4,DNAH1,DYNC1H1,EPPK1,SPTAN1,SYNE1,LAMA2,SACS
GO:0030056	0.006744109	2.171075393	7	28	2	22149	DST,EPPK1
GO:0099568	0.008593653	2.06582218	313	28	5	22149	DST,DNAH11,PCLO,DNAH1,DYNC1H1
GO:0070852	0.00898511	2.046476581	8	28	2	22149	SPTBN4,SACS
GO:0030286	0.008991551	2.046165373	54	28	3	22149	DNAH11,DNAH1,DYNC1H1
GO:0015630	0.009237711	2.034435621	1436	28	9	22149	CDC27,DST,DNAH11,DNAH1,DYNC1H1,SPTAN1,ESPL1,HERC2,UBR4
GO:0005938	0.010734998	1.96919805	328	28	5	22149	DST,PCLO,SPTBN4,DYNC1H1,SPTAN1
GO:0008091	0.011543247	1.937672014	9	28	2	22149	SPTBN4,SPTAN1
GO:0030424	0.030543432	1.515082167	659	28	6	22149	LRP2,DST,PCLO,SPTBN4,DYNC1H1,SACS
GO:0043005	0.041475525	1.382208106	1355	28	8	22149	LRP2,DST,PCLO,SPTBN4,DYNC1H1,SYNE1,LAMA2,SACS
GO:0030016	0.04693789	1.328476438	240	28	4	22149	TTN,DST,SYNE1,AHNAK
REAC:R-HSA-5083625	0.00129038	2.889282425	16	22	3	11004	MUC16,MUC17,MUC5B
REAC:R-HSA-5083636	0.00129038	2.889282425	16	22	3	11004	MUC16,MUC17,MUC5B
REAC:R-HSA-5083632	0.001564861	2.805524359	17	22	3	11004	MUC16,MUC17,MUC5B
REAC:R-HSA-977068	0.004043976	2.393191457	23	22	3	11004	MUC16,MUC17,MUC5B
REAC:R-HSA-5621480	0.005913915	2.228124932	26	22	3	11004	MUC16,MUC17,MUC5B
HP:0008994	0.004511284	2.345699821	73	17	5	5080	TTN,DYNC1H1,SPTAN1,SYNE1,LAMA2
HP:0001437	0.02476841	1.606101867	103	17	5	5080	TTN,DYNC1H1,SYNE1,LAMA2,SACS
HP:0002363	0.030363091	1.517654014	285	17	7	5080	PCLO,DYNC1H1,SPTAN1,SYNE1,DEPDC5,LAMA2,SACS
HP:0001319	0.041405804	1.382938776	196	17	6	5080	DST,PCLO,SPTBN4,HERC2,SYNE1,LAMA2

Supplementary table S9: *Pathway analysis of genes mutated in at least 30% of all MASLD-related tumours; patients with multifocal disease are only represented once.*

Supplementary Table S10: Gene lengths for genes referenced in Table S2A

Gene	Gene size (Mb)
TTN	0.30
ZNF708	0.045
HMCN1	0.46
CDC27	0.071
PCLO	0.41
ZNF726	0.030
DEFB126	0.0032
ZNF430	0.039
ZNF595	0.035
MUC16	0.21

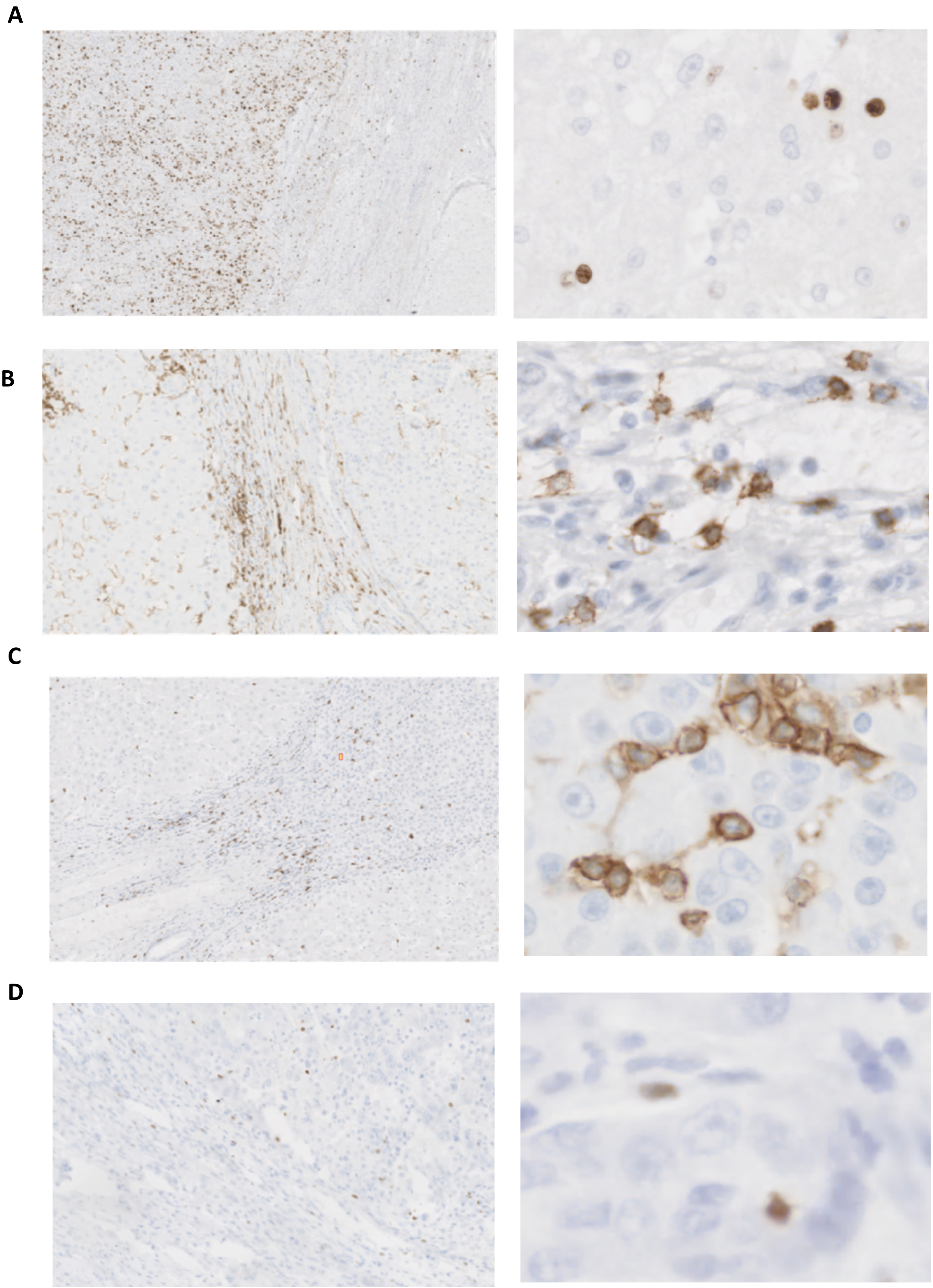

Supplementary Figure S1: **Representative IHC images** at low and high magnification. **(A)** Ki-67, **(B)** CD4+ T cells, **(C)** CD8+ T cells and **(D)** FoxP3+ T cells. Brown DAB staining identifies positive cells.

$$Core = C$$

$$Intermediate = B - C$$

$$Periphery = A - (B + C)$$

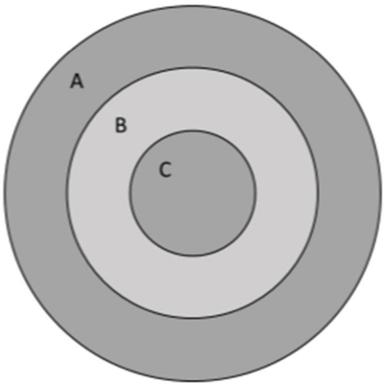

Supplementary Figure S2: Concentric image analysis. Diagram to show overlapping concentric regions created on virtual slides and the equations used for calculating regional Ki-67 positivity.

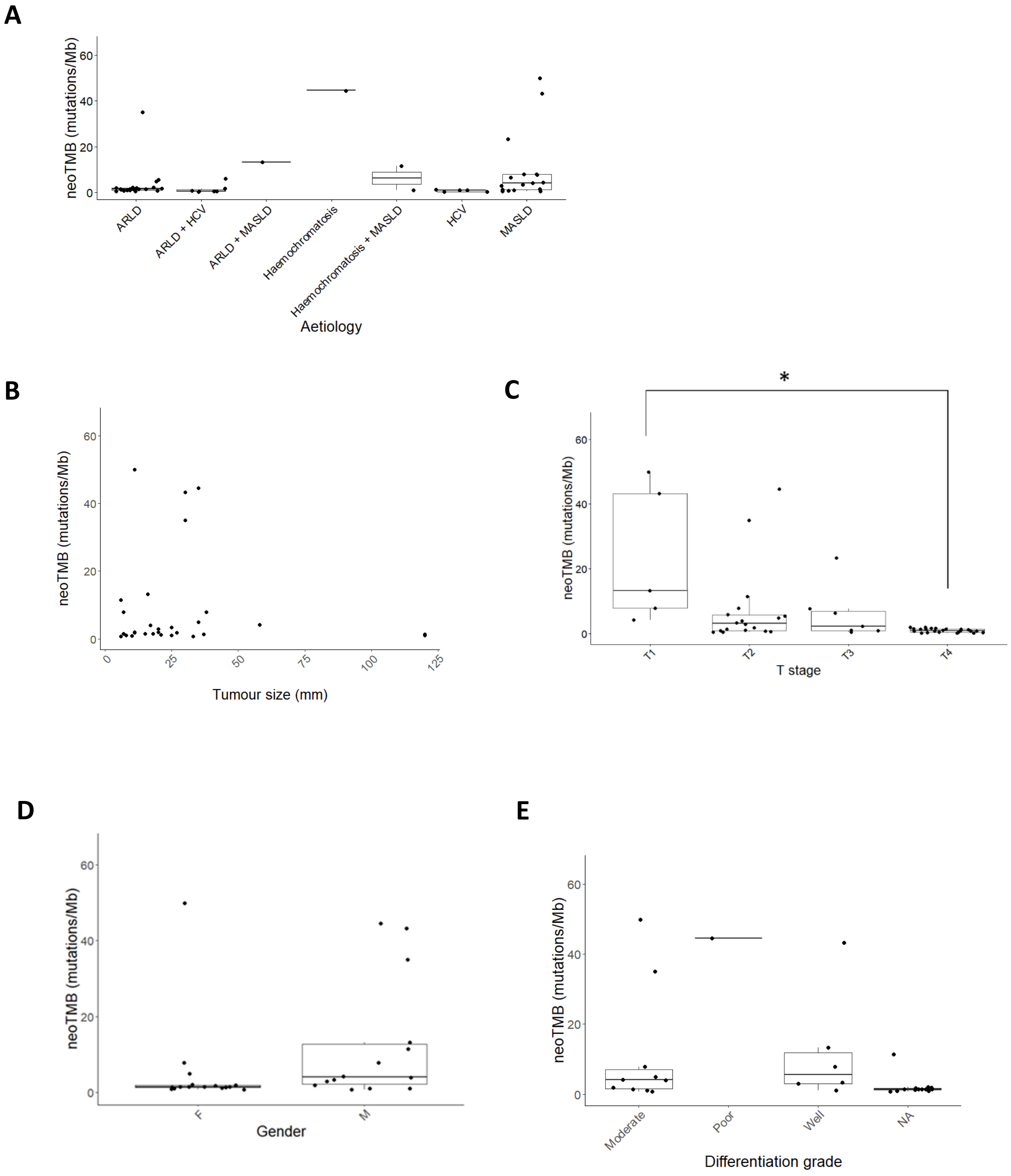

Supplementary Figure S3: neoTMB by patient characteristics

NeoTMB in relation to **(A)** aetiological group, **(B)** tumour size, **(C)** tumour stage **(D)** gender and **(E)** differentiation grade. Adjusted-P value represents a one-way ANOVA with Tukey correction performed (* = < 0.05). N = 30 to 52, lines represent median values.

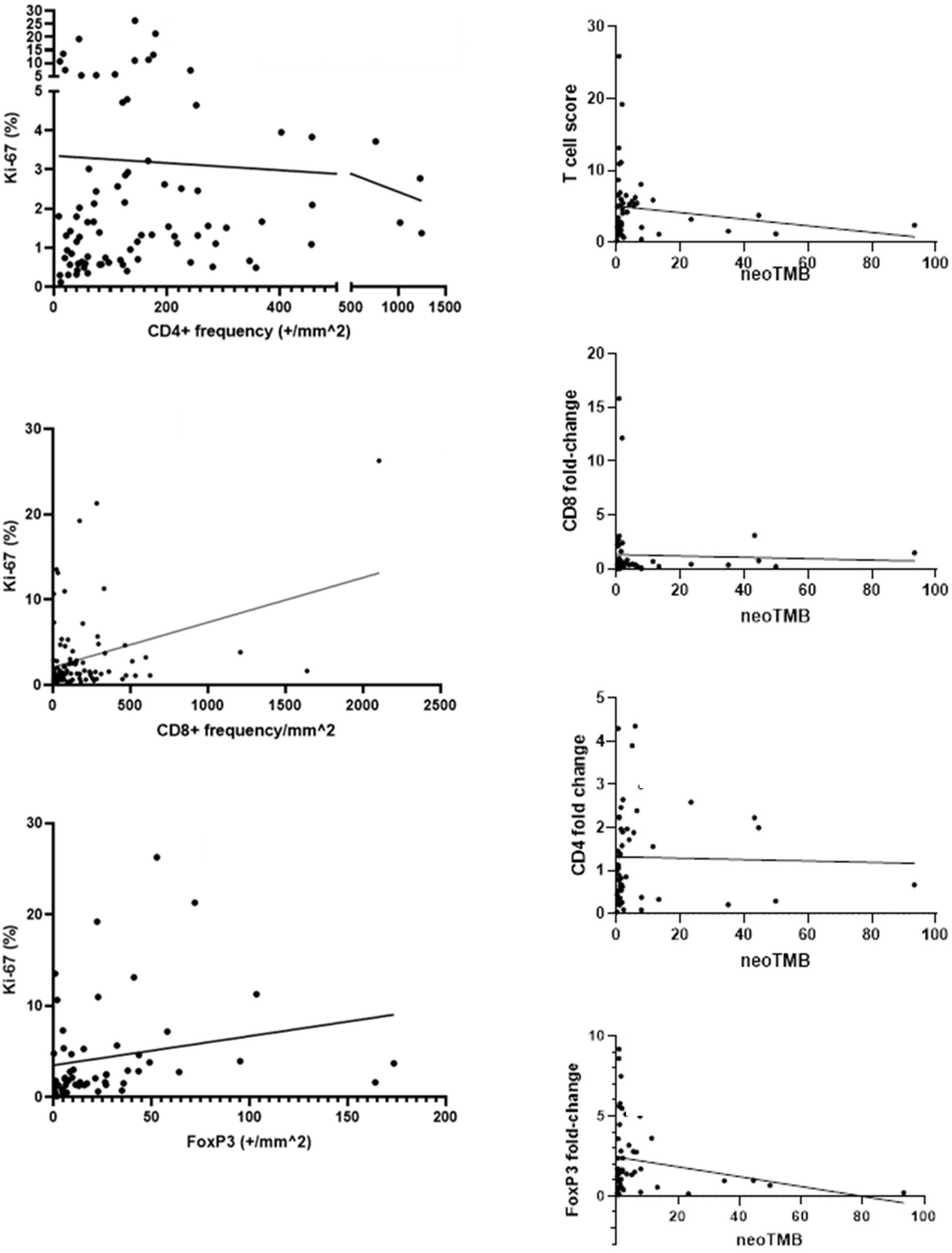

Supplementary Figure S4: *Relationship between neoTMB and Ki-67 or T cell frequencies*.

Scatter plots show tumour-level relationships between neoTMB and**(A)** Ki-67 positivity,**(B)** CD8+ T-cell frequency,**(C)** CD4+ T-cell frequency and**(D)** FoxP3+ T-cell frequency. Associations were assessed using two-tailed Spearman rank correlation. Spearman's correlation coefficients, bootstrap 95% confidence intervals, P values and sample sizes are reported in Supplementary **Table S3**. Missing observations were excluded on a comparison-wise basis. The visual trend line is illustrative only.

Supplementary Table S11. Exploratory adjusted Cox regression.

Univariable Cox proportional-hazards regression were used to examine associations between recurrence-free survival and tumour-to-background FoxP3+ T-cell enrichment, TNM stage and tumour number. TNM stage was analysed as an ordinal variable. Hazard ratios (HR), 95% confidence intervals (CI) and two-tailed *P* values are reported. Assessment using scaled Schoenfeld residuals identified no evidence of violation of the proportional-hazards assumption for the univariable FoxP3 model or either adjusted model.

**Model**	**Variable**	**HR**	**95% CI lower**	**95% CI upper**	***P* value**
Univariable: FoxP3	FoxP3	1.56	1.15	2.13	0.00454
Univariable: tumour number	Tumour number	1.02	0.80	1.3	0.888
Univariable: TNM stage	TNM stage	1.93	1.04	3.61	0.0381
Adjusted: FoxP3 + TNM stage	FoxP3	1.59	1.13	2.24	0.00717
Adjusted: FoxP3 + TNM stage	TNM stage	1.86	0.96	3.59	0.0638
Adjusted: FoxP3 + tumour number	FoxP3	1.56	1.15	2.13	0.00452
Adjusted: FoxP3 + tumour number	Tumour number	0.99	0.77	1.29	0.95

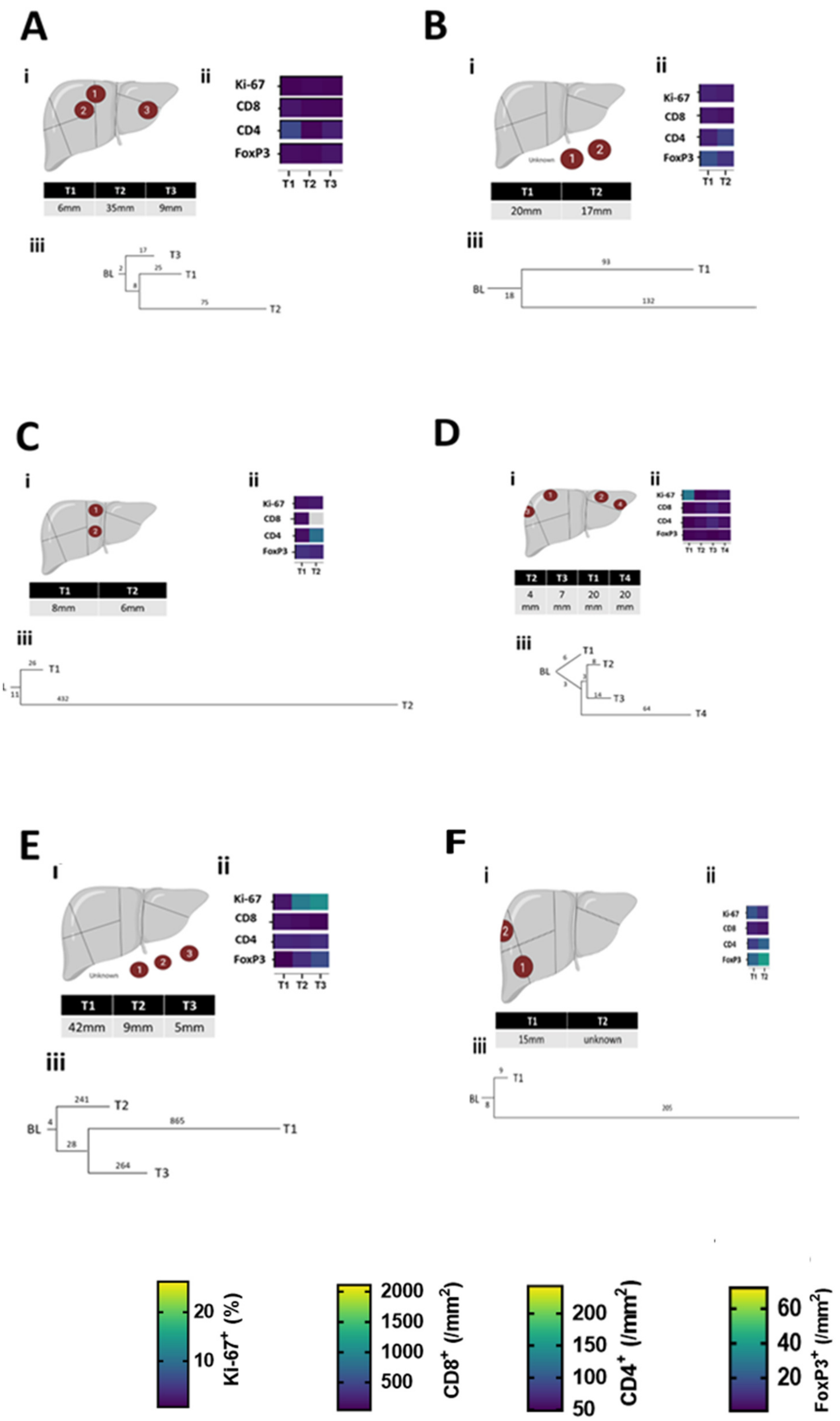

Supplementary Figure S5: *Characterisation of multifocal tumours*

Multifocal patients (A-H) represented with a diagram of the tumour location (i); quantification of Ki-67, CD8+, CD4+, FoxP3+ T cell immunohistochemistry (ii) and phylogenetic trees representing genetic similarity of tumours (iii). Scale bar for immunohistochemistry staining shown in I.

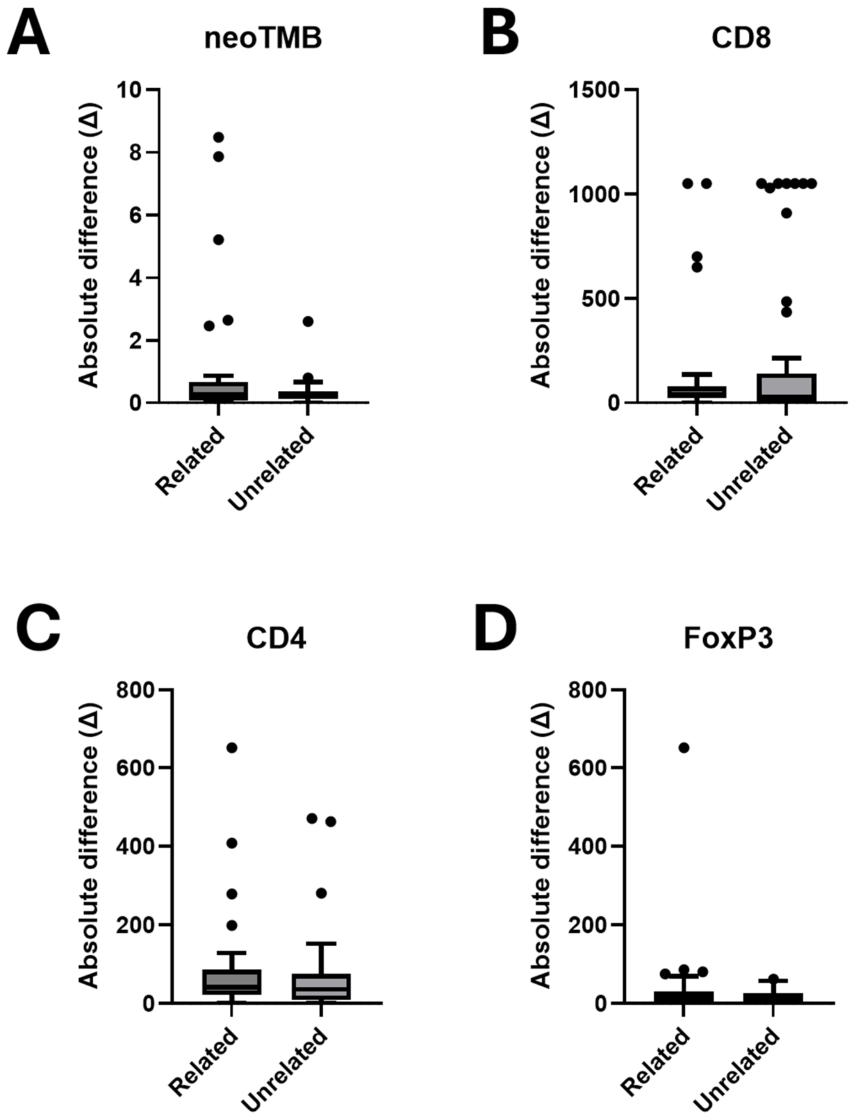

Supplementary Figure S6. Quantitative assessment of inter-lesional heterogeneity

Absolute pairwise differences (Δ) between tumour pairs from patients with multifocal HCC, stratified according to clonal relatedness. Pairwise differences were calculated for**(A)** neoTMB,**(B)** CD8+ T-cell frequency,**(C)** CD4+ T-cell frequency and**(D)** FoxP3+ T-cell frequency. Each point represents a comparison between two tumours from the same patient. Tumour pairs were classified as clonally related or unrelated based on phylogenetic analysis of somatic variants. P vales all > 0.05. Mann-Whitney test.

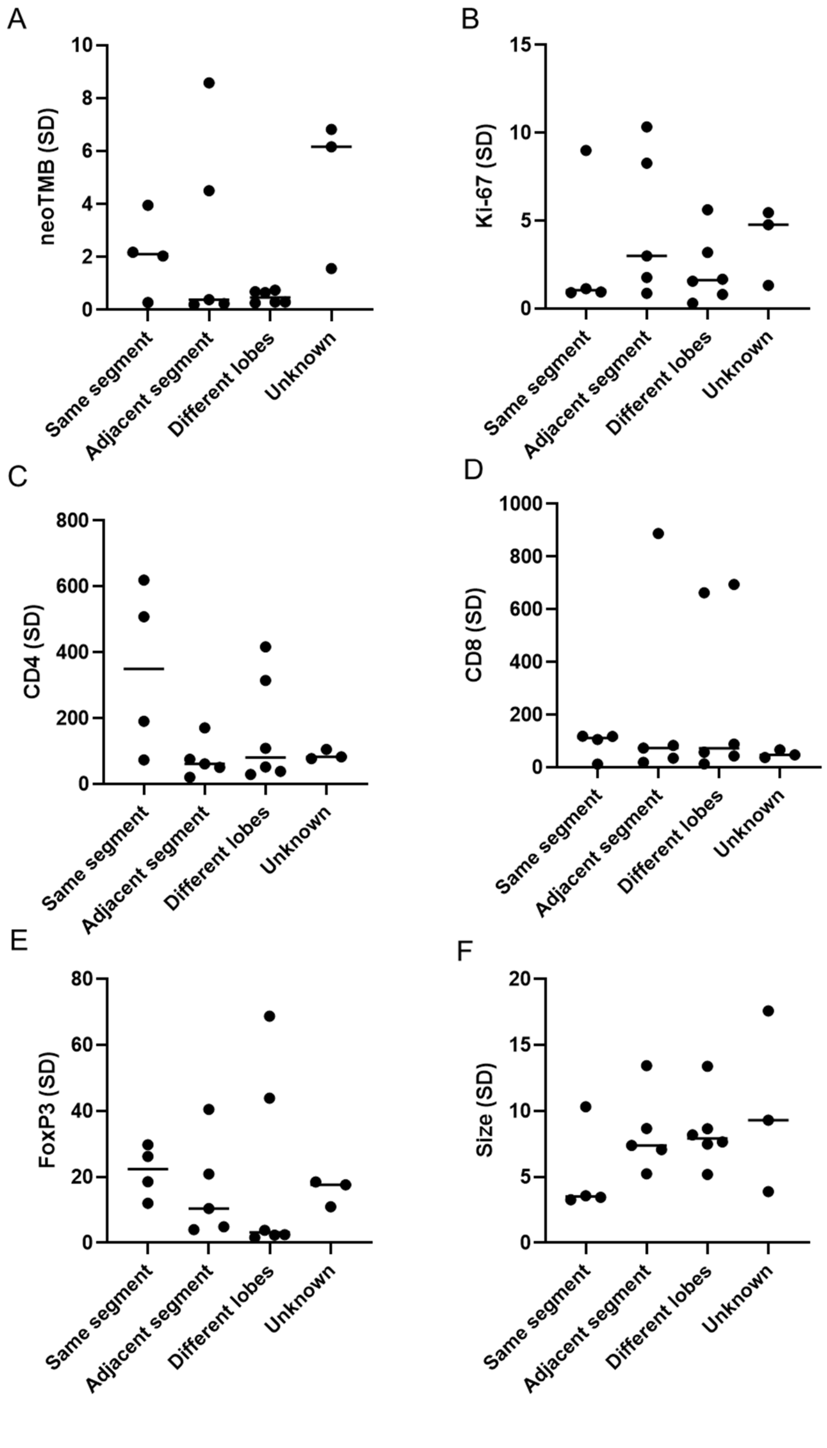

Supplementary Figure S7: Differences according to segmental location

(A) neoTMB, **(B)** Ki-67 positivity, **(C)** CD4+ T cells, **(D)** CD8+ T cells, **(E)** FoxP3+ T cells and **(F)** tumour size. Distance is categorised by two tumours being recorded in the pathology report as being in the same segment, immediately neighbouring segments (adjacent), in the opposite lobe (different lobe) or marked as unknown if the segmental location was not recorded. SD = standard deviation.
